# Advancements in clinical aspects of targeted therapy and immunotherapy in breast cancer

**DOI:** 10.1186/s12943-023-01805-y

**Published:** 2023-07-06

**Authors:** Feng Ye, Saikat Dewanjee, Yuehua Li, Niraj Kumar Jha, Zhe-Sheng Chen, Ankush Kumar, Vishakha, Tapan Behl, Saurabh Kumar Jha, Hailin Tang

**Affiliations:** 1 State Key Laboratory of Oncology in South China, Sun Yat-Sen University Cancer Center, Guangzhou, China; 2 Advanced Pharmacognosy Research Laboratory, Department of Pharmaceutical Technology, Jadavpur University, Kolkata, 700032 India; 3 Department of Medical Oncology, the First Affiliated Hospital, Hengyang Medical School, University of South China, Hengyang, China; 4 Institute of Pathogenic Biology, Hengyang Medical College, University of South China, Hengyang, China; 5 Department of Biotechnology, School of Engineering and Technology, Sharda University, Greater Noida, India; 6 School of Bioengineering & Biosciences, Lovely Professional University, Phagwara, 144411 India; 7 Department of Pharmaceutical Sciences, College of Pharmacy and Health Sciences, St. John’s University, New York, 11439 USA; 8 Pharmaceutical and Health Sciences, Career Point University, Hamirpur, Himachal Pradesh India; 9 School of Health Sciences and Technology, University of Petroleum and Energy Studies, Bidholi, Dehradun, Uttarakhand India; 10 Department of Biotechnology Engineering and Food Technology, Chandigarh University, Mohali, 140413 India; 11 Department of Biotechnology, School of Applied & Life Sciences (SALS), Uttaranchal University, Dehradun, 248007 India

**Keywords:** Breast cancer, Clinical trials, Immune-checkpoint Inhibitors, Immunotherapy, Targeted therapies

## Abstract

Breast cancer is the second leading cause of death for women worldwide. The heterogeneity of this disease presents a big challenge in its therapeutic management. However, recent advances in molecular biology and immunology enable to develop highly targeted therapies for many forms of breast cancer. The primary objective of targeted therapy is to inhibit a specific target/molecule that supports tumor progression. Ak strain transforming, cyclin-dependent kinases, poly (ADP-ribose) polymerase, and different growth factors have emerged as potential therapeutic targets for specific breast cancer subtypes. Many targeted drugs are currently undergoing clinical trials, and some have already received the FDA approval as monotherapy or in combination with other drugs for the treatment of different forms of breast cancer. However, the targeted drugs have yet to achieve therapeutic promise against triple-negative breast cancer (TNBC). In this aspect, immune therapy has come up as a promising therapeutic approach specifically for TNBC patients. Different immunotherapeutic modalities including immune-checkpoint blockade, vaccination, and adoptive cell transfer have been extensively studied in the clinical setting of breast cancer, especially in TNBC patients. The FDA has already approved some immune-checkpoint blockers in combination with chemotherapeutic drugs to treat TNBC and several trials are ongoing. This review provides an overview of clinical developments and recent advancements in targeted therapies and immunotherapies for breast cancer treatment. The successes, challenges, and prospects were critically discussed to portray their profound prospects.

## Introduction

Despite ongoing medical advances, breast cancer remains the second most widespread and fatal malignancy in females [1]. During the past four decades, breast cancer incidences have risen alarmingly [2]. In 2020, there were approximately 2.3 million new cases of breast cancer worldwide, and there were also roughly 6,85,000 deaths from this illness, with notable geographic variations between various countries and regions [3]. Notably, high-economic nations represent a greater percentage of breast cancer fatalities. By 2040, it has been anticipated that there will be more than 3 million new instances of breast cancer each year with more than 1 million annual deaths [4]. There are several different forms of breast carcinoma. It has been found that steroid hormone receptors (HRs) are significant prognostic factors for breast cancer [5]. Breast cancer cells with HRs expressing estrogen (ER), progesterone (PR), or both are referred to as ER-positive (ER +), PR-positive (PR +), or ER/PR positive (ER + /PR +) breast cancers, respectively. Most breast carcinomas are ER + and of those, more than half are both ER + /PR + ; while, only about 2% are solely PR + . Triple-negative breast cancer (TNBC) refers to the cancerous cells lacking ER and PR as well as making too little or no expression of human epidermal growth receptor 2 (HER2). About 10–15% of all breast carcinomas are TNBC. In recent years immunogenic prospects of breast tumor have been revealed. The discovery of tumor-infiltrating lymphocytes (TILs) within breast cancers demonstrated the immunogenic character of breast cancer [6, 7]. Analysis of breast tumor samples revealed that individuals with HER2-positive (HER2 +) and TNBC had a greater number of TILs than those of HR-positive (HR +) subtypes [8, 9].

After decades of endocrine and other therapies, an improved understanding of immune evasion by cancerous cells and the creation of specific immune checkpoint antagonists have unveiled new therapeutic options [10–14]. Immunotherapy involves the enhancement of host immunity and admits cancer as a foreign antigen, which leads to the destruction of cancer cells. However, some adverse events have been advocated in the patients who received immunotherapies, which include fatigue, nausea, vomiting, dizziness, and pruritus [15]. Enterocolitis, endocrinopathies, liver anomalies, and uveitis are some additional immune-related side effects that can result from using immunotherapeutics like ipilimumab. Immune checkpoint blockers are being used in conjunction with chemotherapy, targeted therapy, and radiation therapy under intense examination due to the low efficacy of previous immunotherapeutic drugs in treating many kinds of breast carcinoma. On the other hand, breast cancer-targeted therapies use substances or drugs to inhibit disease progression by obstructing certain components essential for cancer cell survival, proliferation, and migration, as well as for angiogenesis [16]. Poly (ADP-ribose) polymerase (PARP), cyclin-dependent kinase (CDK) 4 and 6 (CDK4/6), Ak strain transforming (AKT), and fibroblast growth factor receptor (FGFR) are well-established therapeutic targets that have been the main focus of drug development for the treatment of breast cancer [17–20]. Patients under targeted therapy also deal with some common side effects such as nausea, vomiting, diarrhea, tiredness, and rashes. Thus, adequate attention must be given to address the toxicity concerns of both the targeted and immune therapies. This review will give a concise overview of the different targeted and immunotherapies used to treat breast cancer. The effectiveness and safety of immunotherapy in combination with chemotherapy and/or radiotherapy, HER2-targeted therapy, CDK4/6 inhibitors, angiogenesis inhibitors, PARP antagonists, and other treatments in the clinical setting were also covered in this review.

## Crosstalk pathway in breast cancer

**Fig. 1:**
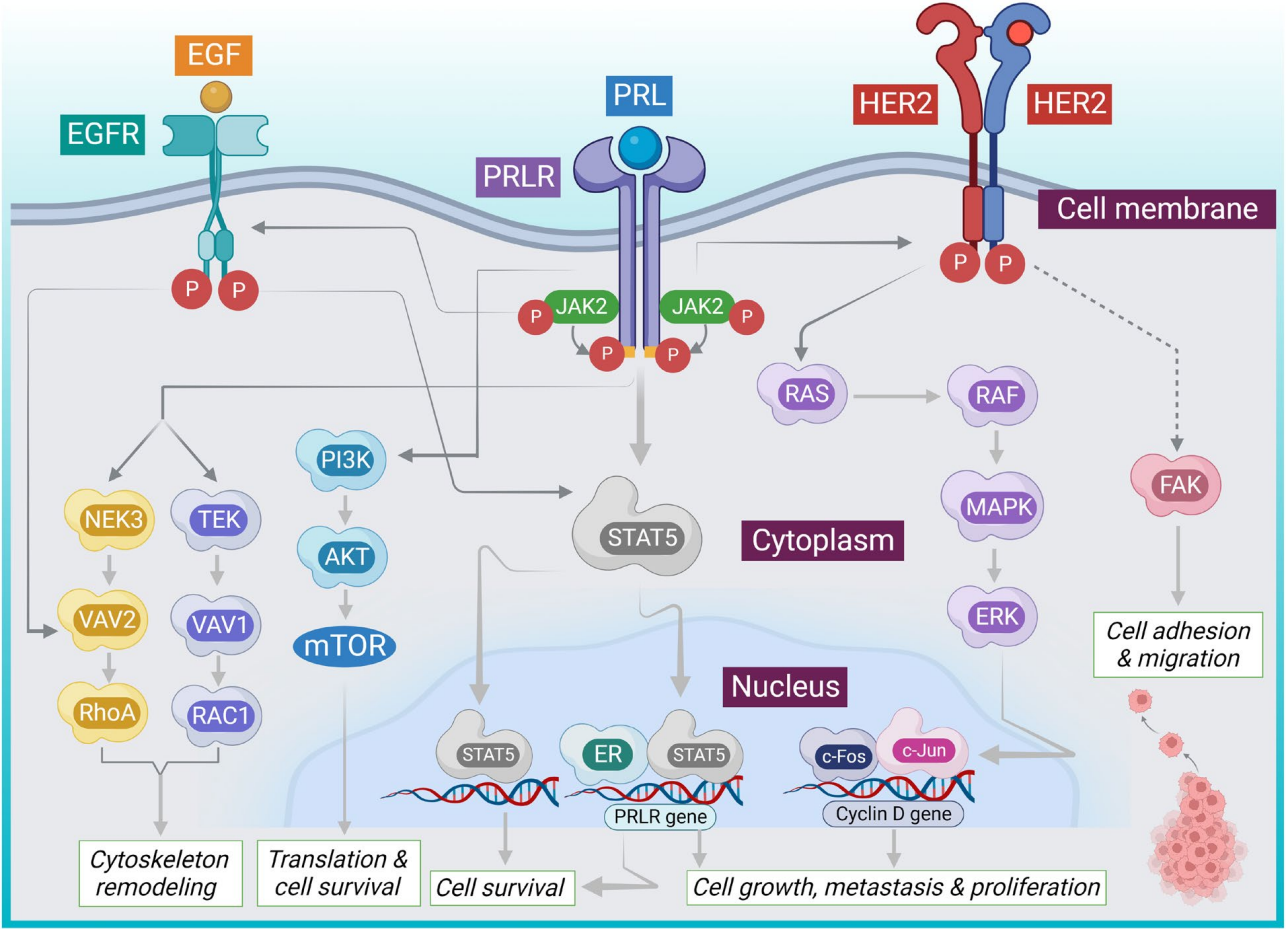
Crosstalk between PRLR and EGFR/HER2 signaling to promote breast cancer progression. PRL endorses PRLR activation, which recruits downstream pathways, such as JAK/STAT, MAPK/ERK, PI3K/AKT/mTOR, NEK3/VAV2/RhoA and TEC/VAV1/RAC1 involved in growth, survival, and migration of breast cancer. EGF/EGFR signaling somewhat overlaps with PRLR signaling to result in the activation of similar downstream events. PRLR/HER2 crosstalk endorses ER phosphorylation and promotes its attachment to the PRLR regulator and promotes PRLR transcription. EGF/EGFR can also trigger PRLR transcription in MAPK/PI3K-dependent manner. In addition, PRL/PRLR activation recruits HRE2 via JAK2 resulting an activation of FAC signaling that promotes cell adherence and induces metastasis. Arrows represent downstream events. AKT, Ak strain transforming; EGF, Epidermal growth factor; EGFR, Epidermal growth factor receptor; ER, Estrogen receptor; ERK, Extracellular signal-regulated kinase; GRB2, Growth factor receptor-bound protein 2; HER2, Human epidermal growth factor-2; JAK, Janus kinase, MAPK, Mitogen-activated protein kinase; mTOR, Mammalian target of rapamycin; NEK3, NIMA-related kinase 3; P, Phosphate; PI3K, Phosphoinositide 3-kinase; PRL, Prolactin; PRLR: Prolactin receptor; RhoA, Ras homolog family member A; STAT, Signal transducer and activator of transcription; TEK, TEK receptor tyrosine kinase; VAV, Vav guanine nucleotide exchange factor 1

A growing body of evidence revealed that the activation of prolactin (PRL) receptor (PRLR) and erythroblastic leukemia viral oncogene homolog receptor (ErbBR) endorses oncogenesis in mammary glands, supports breast tumor growth, and induces chemoresistance [21, 22]. The amplification of the epidermal growth factor (EGF) receptor (EGFR), HER2, as well as PRLR in breast cancers, has been regarded to accelerate tumor growth by activating their downstream signaling pathways [23]. Secretion of PRL from breast cells leads to the activation of downstream PRLR signaling, involving the activation of Janus kinase (JAK)/signal transducer and activator of transcription (STAT), phosphoinositide 3-kinase (PI3K)/Ak strain transforming (AKT), and mitogen-activated protein kinase (MAPK) pathways, which have been implicated in mammary tumorigenesis [24, 25]. When EGF is released, the complete pathway is covered, which aids in the formation of a complex with specificity protein 1 (Sp1) and enhancer binding protein (EBP) resulting in activation of STAT5. Further overexpression of EGFR2 activates the RAS/focal adhesion kinase (FAK) signaling pathways, activating EGFR by cross-phosphorylation. Mitogen-activated protein kinase kinase (MEK)/extracellular signal-regulated kinase 1/2 (ERK1/2) along with PI3K/AKT pathways are jointly induced by PRL and EGF through their corresponding receptors, promoting the proliferation, survival, and metastasis of breast cancer cells [26]. Thus, PRL factors as well as EGFR signaling pathways interact in breast cancer development and progression. In cell line-based assay involving breast cancer cells (MCF-7 and T47D), it has been found that the PRL/PRLR triggers EGFR2 phosphorylation (tyr1221 and 1222) via JAK2, activating the downstream PI3K/AKT signaling [26]. Crosstalk between PRLR and HER2 signaling allows the phosphorylation of ER, its attachment to the PRLR regulator, as well as a rise in PRLR dictation [21]. It has also been revealed that EGF/EGFR could phosphorylate ER (ser118 and 167) and initiate downstream MAPK signaling in breast cancer cells, as well as STAT5b and PRLR transcription [27]. Proto-oncogene tyrosine-protein kinase SRC (c-SRC) endorses EGFR phosphorylation (tyr845) and allows EGFR to indirectly bind to and activate STAT5b. This results in the activation of ER to the generic hPIII promoter and PRLR. It has been revealed that endogenous PRL is necessary for ER on PRLR activation [28]. EGFR can also endorse STAT5 activation directly via phosphorylation in an EGFR-dependent manner [21]. Activated STAT5 translocate to the nucleus, which endorses the transcription of certain genes important for proliferation, diversification, as well as survival. Additionally, it has been revealed that EGFR and HER2 activation are related to STAT3 activation, which also encourages tumor survival and development [21, 28]. Fig. 1 explains the crosstalk between PRLR and EGFR/HER2 signaling to accelerate the growth of breast tumor.

## Targeted therapy

Surgery followed by radiotherapy and chemotherapy is the standard treatment protocol for breast cancer management [29]. The aim of chemotherapy after surgery or radiotherapy is to reduce the likelihood of cancer recurrence. Targeted drug therapy aims at target proteins on breast cancer cells that support their growth, spread, and ability to proliferate [30]. The most effective therapies for breast cancer are those that specifically target the ER and HER2 receptors [31]. A growing body of evidence has proposed some promising novel therapies for several forms of breast cancer, including TNBC and HER2 + carcinomas [32]. Aromatase antagonists, endocrine treatment, selective ER modulators (SERMs), and selective estrogen down regulators (SERDs) are all well-known forms of personalized therapy for HER2 + breast cancer [33, 34]. The course of breast cancer is largely influenced by estrogen and ER [35, 36]. This is the rationale behind the long-standing practice of inhibiting the estrogen signaling pathway in breast cancer patients that are ER + . The first approved drug for treating ER + advanced breast cancer was tamoxifen, which reduces tumor recurrence by roughly 40–50% [37]. SERMs have indeed been utilized to limit cancer growth in estrogen-reliant forms of breast cancer [38]. Inhibitors of any of the targets, such as PARP, HER2, PI3K, AKT, mammalian target of rapamycin (mTOR), fibroblast growth factor (FGF) receptors (FGFRs), and vascular endothelial growth factor (VEGF), could potentially be used as therapeutic approaches to stop the progression of breast cancer due to their roles in various pathways of carcinogenesis, including the cell cycle, angiogenesis, metastasis, etc. [39, 40]. These inhibitors have already demonstrated clinical potential. An mTOR-inhibitor, zortress (42-O-(2-hydroxyethyl)rapamycin) has been authorized for advanced or metastatic aromatase antagonist-resistant ER + breast carcinoma. There is presently no approved targeted therapy for TNBC so far. Given the variety of breast cancer, it is unavoidable that the treatments will be more carefully individualized for each subtype, stage, and grade of breast cancer [41].

### PARP inhibitors

PARP enzymes play crucial roles in many cellular events including the deoxyribonucleic acid (DNA) repairing process [42]. Two enzymes, PARP1 and PARP2, which are activated by DNA strand break, take a role in DNA repair. A growing body of evidence has highlighted PARP as a possible chemotherapeutic target [42]. Inhibition of PARP enzymes prevents DNA repair and causes DNA strands to break, posing a danger to cell survival [43]. Four PARP antagonists have been studied extensively in the clinical setting of breast cancers.

Olaparib, the first US Food and Drug Administration (FDA) approved PARP inhibitor, belongs to N-acyl piperazine class [44]. Olaparib inhibits the activity of DNA topoisomerase 2-binding protein 1 (TOPBP1) and WEE1 (nuclear kinase belonging to the ser/thr family). This results in the buildup of DNA damage that persists during mitosis and resulting in mitotic catastrophe and cell death.. It is one of the PARP inhibitors, which does not cause transaminitis [45]. Importantly, this drug is delivered through the oral route. In the phase III OlympiAD trial (NCT02000622), olaparib (300 mg, twice daily) monotherapy was found to be more effective than standard therapy in patients with metastatic HER2 − breast cancer and germline breast cancer gene (gBRCA) mutations in terms of prolonging progression-free survival (2.8 months) and reducing death (42%) [46]. However, olaparib has been mostly studied as an adjuvant or neoadjuvant in combination with other chemotherapeutic drugs. Olaparib has been used in combination with carboplatin, cyclophosphamide, dacarbazine, durvalumab, eribulin, gemcitabine, paclitaxel, and prexasertib in different clinical studies. In the phase I/II trial (NCT01445418), the olaparib/carboplatin combination is tolerable and exhibits moderate activity in women with sporadic TNBC with the maximum tolerated dose of olaparib was 400 mg, twice daily [47]. However, the majority of grade 3 and 4 adverse events in 36% of patients were neutropenia, followed by thrombocytopenia and anemia in 11% of patients. In another phase I/II trial (UMIN00009498), olaparib (300 mg twice daily)/eribulin combination exhibited antitumor activity against sporadic TNBC patients; though, caution has been recommended if febrile neutropenia is present [48]. 20.8% of patients in phase I and 33.3% of patients in phase II experienced febrile neutropenia. Importantly, PARP inhibition was assured even at the lowest dose of olaparib (25 mg, twice daily). In another phase I/II basket study (NCT02734004), a combination of olaparib (300 mg, twice daily) and durvalumab (1·5 g) exhibited promising anticancer efficacy and was well tolerated in gBRCA-mutated metastatic breast cancer patients [49]. Olaparib given to patients with high-risk, HER2 − early breast cancer and gGRCA-pathogenic or likely pathogenic breast cancer after completion of local treatment was associated with a significantly longer invasive disease-free endurance of patients, according to a phase III trial (NCT02032823) [50]. In a phase II trial (NCT02789332), it was found that individuals with primary HER2 − breast cancer and homologous recombination deficiency responded better to olaparib than carboplatinum as neoadjuvant with paclitaxel in terms of pathological complete response percentage, followed by epirubicin/cyclophosphamide chemotherapy [51]. However, if another drug is being taken that is subject to hepatic metabolism, the use of olaparib must be avoided.

Another potential orally effective PARP inhibitor is talazoparib which is efficacious against gBRCA-mutated, HER2 − breast cancer with an average tolerated dose of 0.60–1 mg/day [52]. In the phase I trial (NCT01286987) involving 18 gBRCA-mutated progressive breast cancer patients, talazoparib (1 mg once daily) monotherapy resulted in a significant therapeutic response rate of 50% and an excellent clinical benefit rate of 86% after 24 weeks of treatment with a good safety and tolerability profile [53]. Talazoparib (1 mg/day) treatment showed promising antitumor activity in a two-stage phase II study (NCT02034916), with manageable adverse effects for gBRCA-mutated progressive breast cancer subjects who had previously undergone platinum-based or non-platinum chemotherapy [54]. In an open-label, randomized, phase III trial (NCT01945775), talazoparib monotherapy significantly outperformed standard single agent-chemotherapy in terms of progression-free endurance among individuals with gBRCA-mutated advanced breast carcinoma [55]. Common side effects were mostly hematologic, rarely happened after medication withdrawal, and could be negotiated with dose adjustments or supportive treatment. In other reports, talazoparib has been revealed to be well tolerated and statistically more effective than other chemotherapeutic drugs in phase III clinical settings [56, 57].

Rucaparib is another orally effective well-tolerated PARP antagonist that ensured sustained PARP inhibition in the surrogate tissues even at the lower doses [58, 59]. In a phase II trial, a lack of objective response rate was seen after intravenous or oral rucaparib treatment within a 21-day cycle in the patients representing gBRCA-mutated locally advanced or metastatic breast tumor. In 39% of breast cancer patients, stable illness on/after 12 weeks was the best outcome [59]. Patsouris and colleagues reported that only a small population of breast cancer patients without gBRCA1/2 mutation represented a significant loss of heterozygosity scores and could be benefited from PARP inhibition by rucaparib [60]. In a phase I clinical trial (NCT01009190), it has been found that oral rucaparib (240 mg/day) can be safely combined with a clinically applicable dose of carboplatin [61]. The clinical observation did not indicate favorable results with cisplatin and low-dose rucaparib combination in BRCA-mutated TNBC [62, 63].

Niraparib is an orally effective PARP inhibitor with effective PARP-trapping activity and has exhibited potential anticancer effects against ovarian and prostate cancer in clinical settings [64, 65]. Niraparib has demonstrated a strong PARP inhibitory impact and improved clinical benefit rates and was well tolerated in patients with gBRAC-mutated locally advanced or metastatic breast cancer [59]. In a randomized, phase III trial (BRAVO), breast cancer patients representing gBRCA mutation were randomized 2:1 between niraparib and the physicians’ optimal chemotherapy [66]. Centrally measured progression-free survival served as the principal outcome. Global survival, progression-free survival by limited evaluation, and objective response rate were considered secondary goals in addition to safety assessment. Following the pre-planned interim investigation, enrollment was stopped due to the occurrence of a significant discrepancy between the local and central progression-free survival assessments in the control (physicians’ choice chemotherapy) arm, which caused informative withholding of the trial [67]. Some trials are ongoing to evaluate the clinical potential of niraparib in breast cancer management.

### CDK4/6 inhibitors

**Fig. 2:**
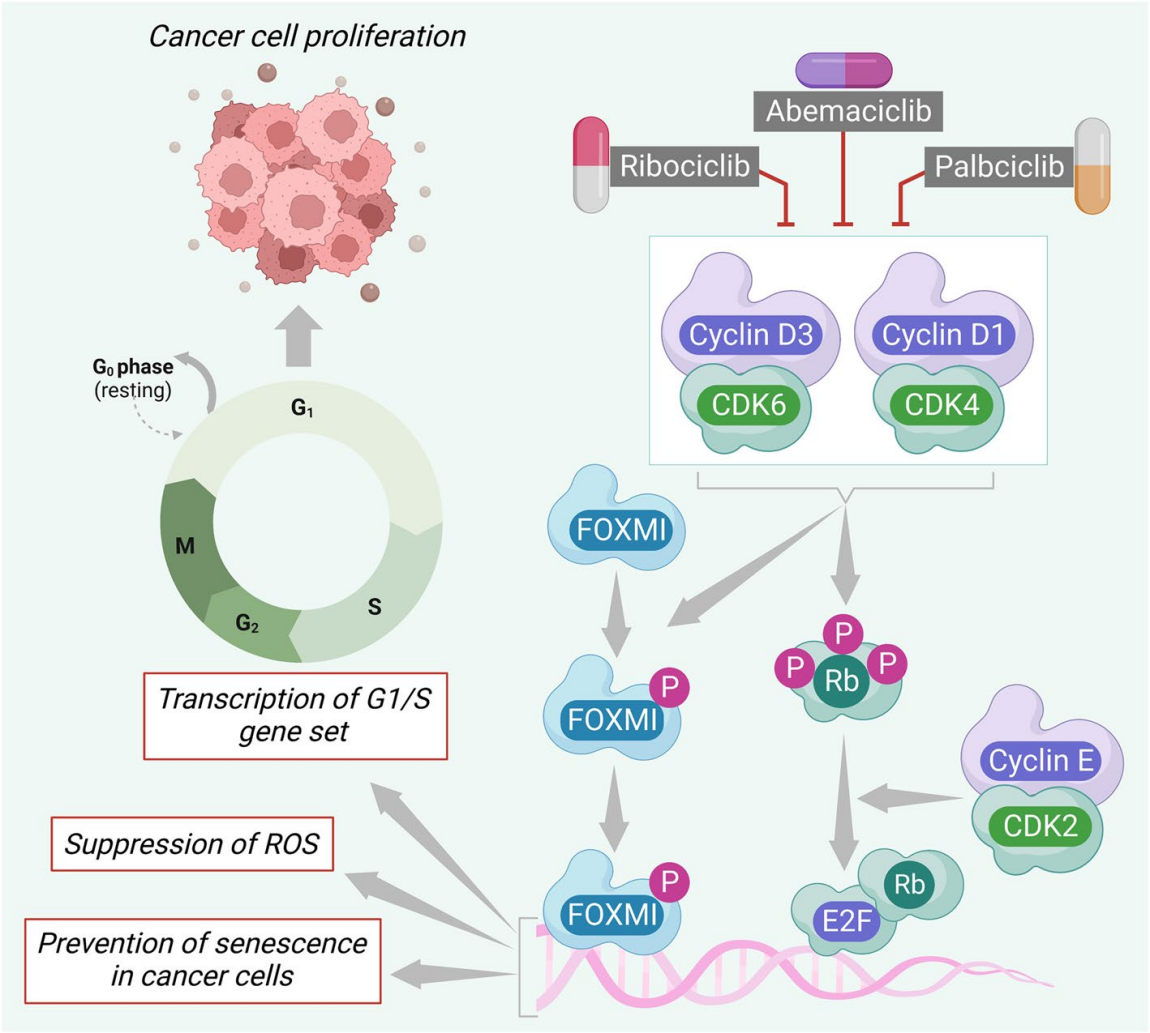
The mechanistic insight of CDK4/6 inhibitors in the management of breast cancer. CDK4/6 activation leads to Rb activation via phosphorylation. Activated Rb enables cell cycle progression by turning on E2F transcription. In addition, CDK4 and CDK6 endorse the stabilization and activation of FOXM1 via phosphorylation, which in turn promotes the upregulation of the G1/S phase genes and the avoidance of cell senescence. CDK4/6 inhibitors, such as palbociclib, ribociclib, or abemaciclib arrest the cell cycle by suppressing CDK4/6 downstream signaling event and arrest. Grey arrows represent downstream events and red lines represent inhibition. CDK, Cyclin-dependent kinase; FOXM1, Forkhead box protein M1; Rb: Retinoblastoma-associated protein

CDK4/6 play important role in the proliferation of cancer cells (Fig. 2). Activation of CDK4/6 by D-type cyclins (cyclin D1/CDK4 and cyclin D3/CDK6) endorses phosphorylation of retinoblastoma-associated protein (Rb) [68]. By turning on E2F transcription, phospho-Rb enables the cell to advance through the cell cycle and divide. Since cyclin D upregulation and restoration phospho-Rb expression is common in HR + breast cancer cells, the G1/S checkpoint is a prime therapeutic target to arrest cancer cell proliferation [69]. In addition, the transcription factor Forkhead box M1 (FOXM1) is an important phosphorylation target of CDK4/6 (Fig. 2). CDK4 and CDK6 endorse the stabilization and activation of FOXM1 via phosphorylation leading to the activation of the G1/S phase gene expressions, the suppression of ROS, and the prevention of senescence in cancer cells [69]. The CDK4/6 inhibitors arrest the cell cycle through this checkpoint (Fig. 2). CDK 4/6 inhibitors are prescription drugs that are clinically effective along with hormonal therapies to treat HR + and HER2 − progressive or metastatic mammary cancer [69]. FDA-approved CDK4/6 antagonists for the treatment of various types of breast cancer include palbociclib, abemaciclib, and ribociclib.

Palbociclib, the first selective orally effective CDK4/6 inhibitor, has received approval for use in treating cancer [70]. It is effective against HR + and HER − breast cancer or metastatic breast carcinoma in pre- or postmenopausal women. It is mainly used in combination with hormonal therapeutics, such as aromatase inhibitor and estrogen receptor antagonist. In contrast, Mayer and colleagues reported that the inclusion of palbociclib did not improve the likelihood of disease-free survival of adjuvant endocrine therapy in phase III clinical settings (NCT02513394) [71]. In phase I trial (NCT00141297), palbociclib exhibited slow absorption (T_max_ = 5.5 h) and elimination (t_1/2_ = 25.9 h) profile with a high distribution volume of 2,793 L. At a dose of 125 mg/day, neutropenia has been identified as the main toxicity [72]. In the phase II trial (UPCC03909; NCT01037790), endocrine-resistant, HR + and Rb + advanced breast cancer patients responded favorably to single-agent palbociclib (125 mg/day, 1–21 days of a 28-day cycle) [73]. Uncomplicated cytopenias have been identified as adverse effects, which are negotiable with dose reduction. In a phase II clinical study (NCT00721409), the inclusion of palbociclib (125 mg/day) to letrozole (2.5 mg/day) showed significant improvement in progression-free survival with progressive ER + and HER2 − breast cancer [74]. Pulmonary embolism, back pain, and diarrhea were recorded as the serious adverse effects of this combination therapy only in a limited number (8%) of patients. Palbociclib was given the FDA approval for the treatment of HR + metastatic breast cancer when used in conjunction with letrozole; studies also indicated that fulvestrant could improve clinical outcomes [75]. In a double-blind, placebo-controlled, randomized phase III trial (NCT01942135), the palbociclib-fulvestrant treatment led to a longer overall survival (though statistically insignificant) than treatment with placebo-fulvestrant among HR + and HER2 − advanced breast cancer patients who had shown sensitivity to prior endocrine therapy [76].

Ribociclib is a highly selective and orally effective CDK4/6 inhibitor used for the treatment of HR + and HER2 − breast cancers with manageable toxicity profiles. In general, ribociclib is administered along with a hormone-blocking agent in breast cancer management [77]. In a phase III trial (NCT01958021), ribociclib (600 mg/day; 1–21 days of a 28-day cycle) in combination with letrozole (2.5 mg/day, continuous treatment) significantly prolonged progression-free survival in postmenopausal women with HR + and HER2 − breast cancers compared with placebo plus letrozole (25.3 months vs. 16.0 months) treated patients [78]. According to a phase III clinical trial (NCT02278120),ribociclib + letrozole treatment improved progression-free survival for premenopausal women bearing HR + and HER2 − progressive breast carcinoma, while exhibited a tolerable safety profile [79].

Abemaciclib is the strongest of these three CDK4/6 inhibitors in terms of CDK4/6 enzyme inhibition potential in vitro. It also demonstrated inhibitory effects on other kinases, such as CDK9 and proto-oncogene serine/threonine-protein kinase (PIM1) [80]. Oral treatment, continuous dosing, effective target inhibition, and manageable toxicities make abemaciclib a new therapeutic option for the most prevalent forms of breast cancer [81]. The highest tolerated dose of abemaciclib was established to be 200 mg, twice daily accordance with a phase I trial (NCT01394016) [82]. Single-agent abemaciclib exhibited a higher clinical benefit rate in HR + breast cancer compared with HR − cases. Compared to monotherapy, combination with fulvestrant slightly improved the clinical benefit rate in HR + breast cancer cases. In an open-label phase II trial (NCT02102490), abemaciclib exhibited potential clinical efficacy and tolerability in HR + and HER2 − metastatic breast cancer management [83]. The FDA approved abemaciclib (200 mg, twice daily) monotherapy on September 28, 2017, for women with HR + progressive or metastatic breast cancer continuing on both hormone treatment and chemotherapy, despite substantial dose reduction or omission rates were seen in phase II trial at this dose range [81, 83]. Abemaciclib in combination with endocrine therapy with antiestrogenic drugs in breast cancer management has been found to be efficacious with manageable safety profile in clinical settings. In the phase III double-blind trial (NCT02107703), abemaciclib (150 mg, twice daily) in combination with fulvestrant was efficacious, dramatically enhancing progression-free survival and objective response rate and exhibiting a tolerable safety profile in women with HR + and HGR2 − progressive breast cancer who proceeded while receiving hormone therapy [84]. A similar observation has been recorded with abemaciclib (150 mg, twice daily) in combination with a non-steroidal aromatase inhibitor either anastrozole (1 mg/day) or letrozole (2.5 mg/day) in a phase III clinical study (NCT02246621) involving postmenopausal women with HR + and HGR2 − progressive breast cancer [85]. Abemaciclib plus fulvestrant has been revealed to improve therapeutic outcomes in clinical settings (NCT02107703) for premenopausal women with HR + and HGR2 − progressive breast carcinoma who are resistant to hormone therapy [86].

The toxicity profiles of all three CDK4/6 inhibitors are comparable [81]. The FDA has warned that certain patients with advanced breast cancer who are being treated with CDK4/6 inhibitor, palbociclib, ribociclib, or abemaciclib may experience a rare but serious lung inflammation.

### AKT Inhibitors

**Fig. 3:**
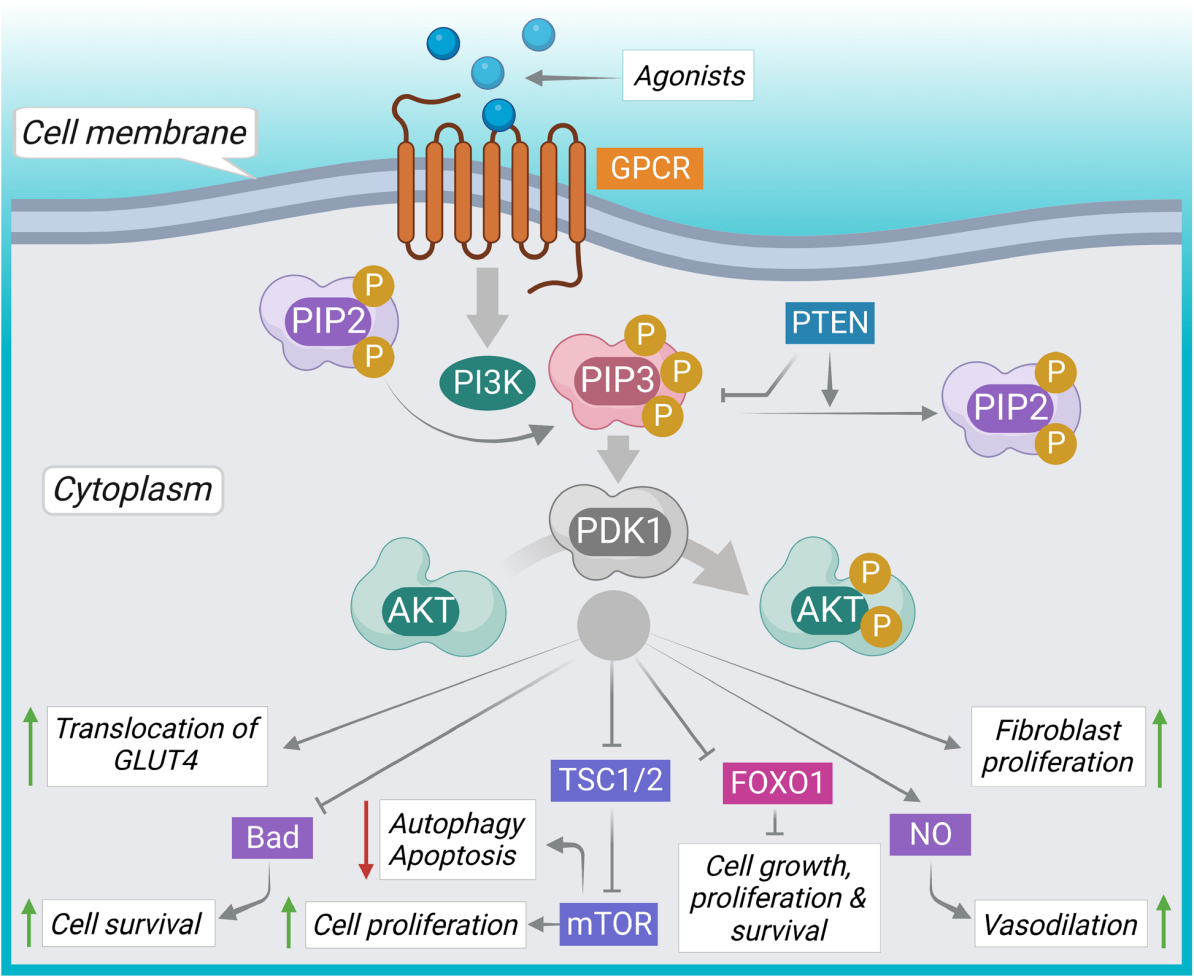
AKT signaling pathway in breast cancer development and progression. It is a major intracellular pathway which leads to cell survival and cell proliferation. Activation of PI3K catalyzes the phosphorylation of PIP2 to PIP3, which further endorses PDK1 activation. Phosphorylation of FOXO1 by AKT inhibits its transcriptional activities resulting in cell growth, proliferation and survival. In addition, AKT inhibits TSC1/2 resulting in an activation of mTOR which simultaneously suppress autophagy and apoptosis and triggers proliferation. Gray arrows represent downstream events, gray lines represent inhibition, green up arrowheads represent activation/upregulation, and red down arrowheads represent suppression/downregulation. AKT, Ak strain transforming; Bad, Bcl-2-associated death promoter; FOXO, Forkhead box transcription factors; GPCR, G protein-coupled receptor; mTOR, Mammalian target of rapamycin; NO, Nitric oxide; PDK1, Phosphoinositide-dependent kinase-1; PI3K, Phosphoinositide 3-kinase; PIP2, Phosphoinositol 4, 5-biphosphate; PIP3, Phosphoinositol 3, 4, 5-triphosphate; PTEN, Phosphatase and tensin homolog is a phosphatase; TSC, Tuberous sclerosis complex

AKT is a crucial transducer in PI3K/AKT/mTOR signaling pathway that promotes the growth, division, migration, survival, and senescence of cells as well as the progression of cancer (Fig. 3) [17, 87]. PI3K is the key component of AKT signaling, which is a heterodimer comprising two subunits, such as p85 (regulatory) and p110 (catalytic). PI3K is activated by different growth stimuli via phosphorylation of the p85 subunit. Upon activation, PI3K endorses PIP2 (phosphoinositol 4, 5-biphosphate) phosphorylation to form PIP3 (phosphoinositol 3, 4, 5-triphosphate) in the plasma membrane, which acts as a secondary messenger of AKT recruitment [88, 89]. Phosphatase and tensin homolog is a phosphatase (PTEN) that acts as a negative regulator of PI3K/AKT signaling. Activation of AKT via phosphorylation endorses phosphorylation of forkhead box O1 (FOXO1) resulting in transcriptional suppression of FOXO1 and inhibition of its proapoptotic role [89, 90]. In addition, activation of mTOR via TSC1/2 phosphorylation is endorsed by AKT resulting in suppression of cell death pathways and activation of cell proliferation. AKT activation is regarded to directly suppress the activation of pro-apoptotic factors like the Bcl-2-associated death promoter (Bad). In addition, mutation of the PI3K catalytic alpha (PIK3CA) gene is known to activate PI3K. PIK3CA gene mutation is abandoned in HR + and HER + breast cancer; while it is less frequent in TNBC. On the other hand, TNBC has been found to be associated with PTEN loss [91]. Eventually, breast cancer cells are associated with activated PI3K/AKT signaling associated with either PIK3CA point mutations or PTEN suppression depending upon the type of breast cancer. Thus, inhibition of PI3K/AKT signaling could be attributed to arresting breast cancer recurrence and can aid a dimension in breast cancer management.

Different ATP-competitive and allosteric AKT inhibitors have been discovered so far and tested in clinical platforms [92]. Among allosteric inhibitors, only MK-2206 has been tested on breast cancer patients. In a phase I study (NCT00963547), a combination of MK-2206 and trastuzumab was well tolerated by patients with HER2 + tumors; however, MK-2206 treatment has been observed to support HER3 activation via feedback mechanisms, reducing anticancer efficacy [93]. In early-phase clinical trials, MK-2206 in combination with other drugs did not show a potential therapeutic response [94, 95]. In the I-SPY 2 trial (NCT01042379), MK-2206 (135 mg/week) and anthracycline- or taxane-based neoadjuvant chemotherapy improved complete pathologic response in HER2 + and/or HR − breast cancer patients [96].

ATP-competitive AKT inhibitors provide a superior therapeutic window than allosteric inhibitors. Two ATP-competitive AKT inhibitors, capivasertib and ipatasertib, were subjected to detailed phase I and II clinical trials as monotherapy or in combination with hormone therapeutics or chemotherapeutic drugs [97]. The first phase I open-label trial (NCT01226316) with capivasertib recommended the oral dose of 480 mg, twice daily (4/7 intermittent) for phase II trial [98]. In this trial, capivasertib showed a reduction in tumor size in PIK3CA-mutant breast cancer patients. In another phase I study (NCT01090960), capivasertib showed effective suppression of AKT signaling and disease control efficacy against solid tumors including breast tumors [99]. In these phase I trials, capivasertib has been well tolerated. In another phase I trial (NCT01226316), AKT1^E17K^-mutant and ER + metastatic breast cancer patients who had received extensive pretreatment with capivasertib alone or in combination with fulvestrant, including those whose disease had previously progressed on fulvestrant, exhibited clinically significant therapeutic efficacy [100]. Moreover, the activity and tolerability seemed to be enhanced by this combination.

Capivasertib and fulvestrant combination has also ensured enhanced progression-free survival of patients with ER + metastatic breast cancer in the phase II clinical setting (NCT01992952) [101]. In a randomized and double-blind phase II trial (NCT02423603), the inclusion of capivasertib with paclitaxel chemotherapy improved progression-free and overall survival of patients with metastatic TNBC [102]. In contrast, in a phase I/II trial (NCT01625286), a combination of capivasertib to paclitaxel failed to show improvement of the chemotherapeutic potential of paclitaxel among the patients with PIK3CA-mutated or overall ER +/HER2 − metastatic/progressive breast cancer [103]. In the phase II trial (NCT02077569), capivasertib (480 mg, twice daily) effectively suppressed primary end-point biomarkers of the AKT signaling pathway, such as phospho-GSK3β and phospho-PRAS40 as well as dropped Ki67, a proliferation marker after 4.5 days treatment in invasive breast cancer patients [104]. In addition, significant changes in the secondary biomarkers namely phospho-AKT, Forkhead box O3 and phospho-S6 were observed following capivasertib treatment. A few phase III trials (NCT03997123 and NCT04305496) to evaluate the clinical efficacy of capivasertib along with chemotherapeutic or anti-estrogenic drugs in different forms of breast cancer are underway. In addition, capivasertib could aid in radiotherapy as a radiosensitizer [105].

Ipatasertib is another orally effective ATP-competitive AKT inhibitor, which has demonstrated potential anticancer activity against solid tumors in both pre-clinical and clinical studies [106]. In the phase II LOTUS trial (NCT02162719), adding ipatasertib to paclitaxel improved the progression-free survival of patients with metastatic TNBC [107]. In another randomized phase II trial (NCT02301988), the inclusion of ipatasertib co-treatment with paclitaxel chemotherapy (12 weeks) did not cause a significant improvement in pathologic complete response rate in early TNBC patients; however, showed a good overall response rate and antitumor effect evidenced by magnetic resonance imaging and biomarker analyses [108]. In contrast, in a double-blind, randomized phase III trial (NCT03337724), the inclusion of ipatasertib to paclitaxel did not improvement of efficacy in terms of progression-free survival among HR + and HER2 − progressive breast cancer patients [109]. It is thought to be targeting ER along with AKT inhibition could offer a better therapeutic output in breast cancer management. Several phase III trials (NCT04650581, NCT04060862 and NCT04177108) are underway to evaluate the clinical efficacy of ipatasertib in combination with other drugs to treat different forms of breast cancer.

### Angiogenesis inhibitors

Angiogenesis is the rapid increase in the formation of blood vessels to ensure sufficient oxygen supply to the tumor cells. The angiogenic switch in cancer cells is regulated by several mechanisms which encourage the growth of new blood vessels and raise the possibility of distant metastasis [110]. A variety of angiogenesis inhibitors can be therapeutically used to treat various types of advanced solid tumors [111]. These inhibitors include monoclonal antibodies or small-molecule tyrosine kinase inhibitors (TKIs), which primarily target the traditional VEGF and its receptors (VEGFRs). In TNBC treatment, angiogenesis inhibitors like VEGFR targeting agents have been the focus of most research [110]. It is important to mention that around 15–20% of all breast cancer patients represent HER2 + malignancy [112]. Increased angiogenesis and the expression of VEGF are strongly correlated with HER2 activation in cancer cells [113]. Thus, HER2 inhibition would serve as a potential treatment approach for the treatment of HER2 + tumors. Several anti-HER2 antibodies and HER2 TKIs have shown therapeutic promise in the management of HER2 + breast cancer.

Anti-angiogenesis monotherapy has a minimum effect on complex TNBC; however, it has been found to have a workable antitumor impact when added to standard chemotherapy [114]. Bevacizumab, an anti-VEGF monoclonal antibody has been studied in clinical settings to inhibit angiogenesis to treat metastatic and proliferative breast cancer. The clinical study (phase III) data demonstrated that adding bevacizumab to capecitabine significantly improved response rates but did not cause improvement in overall survival or progression-free survival of metastatic breast cancer patients [115]. In a randomized, open-label phase III E2100 trial (NCT00028990), the inclusion of bevacizumab along with paclitaxel chemotherapy improved progression-free survival but not overall survival of metastatic breast cancer patients compared to paclitaxel monotherapy [116]. Considering the outcome of this E2100 trial, the FDA approved bevacizumab for the treatment of breast cancer [117]. In the E2100 trial, Schneider and colleagues found a correlation between the VEGF genotype and overall survival as well as grade 3/4 hypertension in patients with metastatic breast cancer using bevacizumab. The result of the E2100 trail was further validated through several phase III clinical trials viz. AVADO (NCT00333775), RIBBON-1 (NCT00262067), and RIBBON-2 (NCT00281697). Overall survival was not improved in breast cancer patients in any of them, and progression-free survival was shown to be less than in the E2100 study. In the phase III AVEREL trial (NCT00391092), locally recurrent or metastatic HER2 + breast cancer patients treated with bevacizumab in conjunction with trastuzumab and docetaxel did not show significant improvement in progression-free survival [118]. Meta-analyses of randomized trials showed that the inclusion of bevacizumab with chemotherapeutic drugs improves the overall and progression-free survival of progressive and metastatic breast cancer patients [119–121]. Ramucirumab, an anti-VEGFR2 monoclonal antibody has been approved by the FDA in 2014 for the treatment of metastatic gastric cancer. A few phase II (NCT01234402 and NCT01427933) and III (NCT00703326) trials have been undertaken, where ramucirumab alone or in combination with other therapeutic drugs did not show any promising therapeutic outcome in breast cancer management [122]. Moreover, adding ramucirumab to docetaxel treatment has been found to increase the risk of toxicity in patients with metastatic breast cancer [123].

TKIs are a class of compounds that target the catalytic roles of the VEGFRs and other growth factor receptors associated with angiogenesis [124]. Sunitinib, sorafenib, axitinib, pazopanib, cediranib and vandetanib are the TKIs that have been studied in the clinical setting of breast cancer. Among them, cediranib (NCT00454805) and vandetanib (NCT00880334) exhibited poor therapeutic efficacy in phase II trials when combined with fulvestrant and docetaxel, respectively [125, 126]. Axitinib also did not show significant therapeutic output to metastatic breast cancer patients when combined with docetaxel in a double-blind, randomized phase II clinical trial (NCT00076024) [127]. Pazopanib, an orally effective multitarget TKI exhibited promising therapeutic efficacy in terms of disease stability in recurrent or metastatic invasive breast cancer patients with allowable untoward effects in the phase II clinical setting [128]. However, combining pazopanib with other anticancer drugs either did not demonstrate a potential therapeutic response or produced unacceptable toxicity [122]. Sorafenib, an orally effective multitarget kinase inhibitor has been approved for the treatment of different types of cancer. However, sorafenib demonstrated mixed observations in the clinical setting of breast cancer when combined with other drugs. In a double-blind, randomized phase II trial (NCT00493636), the combination of sorafenib with gemcitabine or capecitabin promoted progression-free survival moderately in HER2 − breast cancer patients with manageable toxicity [129]. In contrast, in some phase II (EudraCT ID 2007–000290-32) and phase III (NCT01234337) trials, the inclusion of sorafenib to capecitabin therapy did not improve survival advantage as well as imparted incidences of toxicities in HER2 − breast cancer patients [130, 131]. Similarly, other trials did not reveal any therapeutic benefit of sorafenib along with standard chemotherapy in the management of different forms of breast cancer [132]. Sunitinib is another multitarget kinase inhibitor that was approved by the FDA for the treatment of advanced renal cell carcinoma, pancreatic neuroendocrine, and gastrointestinal stromal cancers. Some clinical studies have been undertaken to see the effect of sunitinib in breast cancer management. However, in the clinical setting of breast cancer, sunitinib monotherapy or combination with other drugs failed to establish any promising outcome.

### FGFR inhibitors

**Fig. 4:**
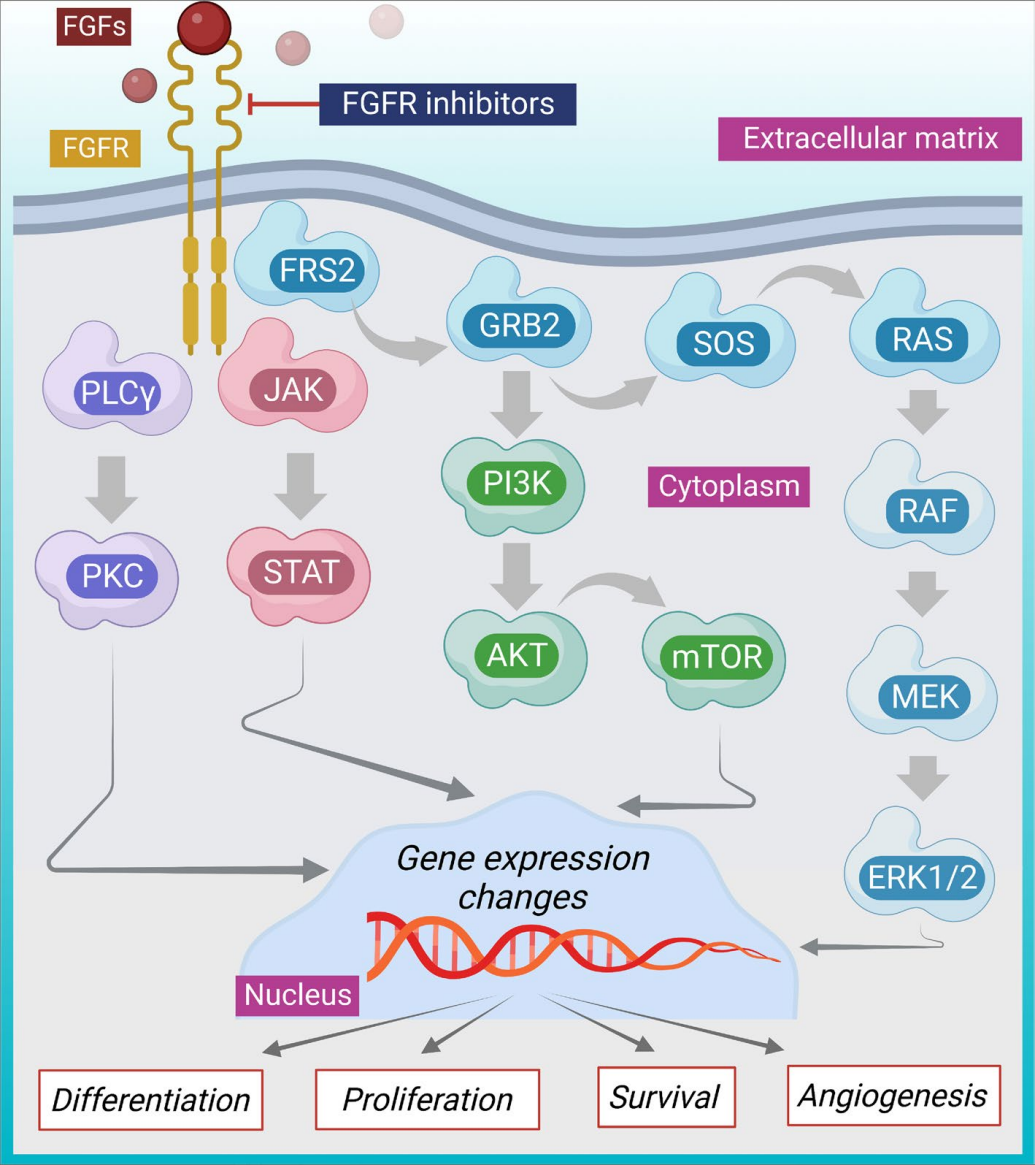
FGFR signaling pathway in breast cancer as a potential therapeutic target. Attachment of FGFs to FGFRs causes their dimerization, which encourages TGFR1 activation through kinase domain activation loop with activation of FRS2, PLCγ and downstream transduction pathways, such as PI3K/AKT/mTOR, PKC, RAS/MAPK pathways, which potentiate proliferation, differentiation, migration, angiogenesis, and survival process. Arrows represent downstream events and the line represents inhibition. AKT, Ak strain transforming; ERK, Extracellular signal-regulated kinase; FGF, Fibroblast growth factor; FGFR, Fibroblast growth factor receptor; FRS2, Fibroblast growth factor receptor substrate 2; GRB2, Growth factor receptor-bound protein 2; JAK, Janus kinase; MEK, Mitogen-activated protein kinase kinase; mTOR, Mammalian target of rapamycin; PI3K, Phosphoinositide 3-kinase; PKC, Protein kinase C; RAF, Rapidly accelerated fibrosarcoma; RAS, Rat sarcoma; SOS, Son of sevenless; STAT, signal transducer and activator of transcription

FGF/FGFR constitute an important signaling network involved in the regulation of growth, survival, differentiation, migration, and apoptosis of cancer cells (Fig. 4) [133]. When FGFs attach to FGFRs, the receptors become dimerized, which causes TGFR1 transphosphorylation through a kinase domain activation loop. Following this, FGFR substrate 2 (FRS2) and phospholipase C, two intracellular receptor substrates are activated via phosphorylation. Furthermore, active FRS2 encourages downstream signaling via the PI3K/AKT and/or RAS/MAPK pathways, which control cell proliferation, differentiation, as well as survival [134, 135]. Genetic shifts in FGFRs may lead to the development of tumors and unfavorable outcomes in women with breast carcinoma [19]. The FGFR system is frequently dysregulated in various cancers, however, most of the research focuses on FGFR1, 2, and 3. FGFR4 is also implicated in oncogenesis, tumor growth, and resistance to anti-tumor treatment in different types of cancer. A cohort of 391 tumor patients revealed that FGF/FGFR abnormalities are common in breast cancer (32.1%) and frequently co-exist with other abnormalities [19]. For instance, specific anomalies linked to changes in FGF/FGFR were found in 15 genes in a univariate analysis. Unusual activation FGF/FGFR axis has been revealed in various clinical subtypes of breast cancer, thus inhibition of FGFR would serve as an approach to cancer management.

Among FGFR inhibitors, TKIs are important. The therapeutic roles of some important non-selective TKIs on breast cancer patients have been discussed in the earlier section. Dovitinib, lucitanib, and lenvatinib are some additional members of non-selective TKIs, which have been subjected to clinical trials in breast cancer setting. Dovitinib in association with fulvestrant showed promising clinical efficacy to ER + or HER2 + breast cancer in postmenopausal women in a phase II clinical setting (NCT01528345); however, the trial was discontinued in response to the poor enrollment of FGF-expressing patients [136]. Lucitanib is another orally effective and multitarget TKI that has been subjected to a phase II clinical study (NCT02202746) involving patients with/without FGFR1-amplified metastatic breast cancer. Lenvatinib is now being tested in a phase II clinical trial (NCT03168074) for the treatment of patients with early-stage ER + breast cancer.

To understand the on-target FGFR inhibition in patients with FGF/FGFR anomalies, selective FGFR inhibitors have been developed. Among selective FGFR inhibitors, infigratinib, erdafitinib, AZD4547, Debio-1347, and TAS-120 have been subjected to clinical trials in breast cancer settings. In the phase I clinical trial (NCT01928459), the combination of infigratinib (125 mg/day), a pan-FGFR inhibitor, with alpelisib, a PIK3CA inhibitor failed to reveal any conclusive evidence of synergistic effect in the patients with PIK3CA-mutant progressive solid tumors (including metastatic breast cancer), with/without FGFR/2/3 amplifications [137]. In the phase II trial (NCT01795768), AZD4547, an FGFR1/2/3 inhibitor exhibited a potential therapeutic effect on FGFR1-amplified breast tumor patients [138]. A phase II clinical trial (NCT01791985) involving patients with ER + breast cancer has been carried out to assess the therapeutic potential of AZD4547 when combined with letrozole or anastrozole, however, the results are not yet available. The trials with Debio-1347 (NCT03344536), TAS-120 (NCT04024436) and erdafitinib (NCT03238196) on patients bearing different subtypes of breast cancer are ongoing.

Although FGFR antagonists has received a lot of attention, evaluating the efficacy of monoclonal antibodies or ligand-trapping agents has been simultaneously taken into consideration to achieve anti-FGFR effect. GP369, FPA144, and MFGR1877S are a few monoclonal antibodies that have been developed to block FGFR2-IIIb, FGFR2, and FGFR, respectively. FP-1039 (GSK3052230), a ligand trap has been developed to sequester FGFs and thus inhibits FGFR signaling. In a phase I trial (NCT00687505), FP-1039 exhibited good tolerability and acceptable safety profile on solid tumor (including breast tumor) patients. However, more clinical research is required to develop them as drug candidates for breast cancer management. Table 1 shows some important FDA-approved small molecules for targeted therapy of breast cancer.

**Table 1:** List of FDA-approved small molecules for targeted therapy of breast cancer

S. no	Drugs (Year of regular approval)	Structures	Breast cancer subtypes	Conditions	Comments	References
**PARP inhibitors**						
1	Olaparib (2018)		gBRCA-mutated HER2 − metastatic BC	Combination with chemotherapy in the adjuvant and neoadjuvant setting	Approval was based on the phase III OlympiAD trial (NCT02000622)	[129]
2	Talazoparib (2018)		gBRCA-mutated HER2 − locally advanced or metastatic BC	**-**	Approval was based on the phase III EMBRACA trial (NCT01945775)	[52]
**PI3K inhibitor**						
3	Alpelisib (2019)		HR + /HER2 − , PIK3CA-mutated, advanced or metastatic BC	Combination with an ER antagonist, fulvestrant	Approval was based on the phase III SOLAR-1 trial (NCT02437318)	[139]
**mTOR inhibitor**						
4	Everolimus (2012)		Postmenopausal women with advanced HR + /HER2 − BC patients who have relapsed/ progressed following treatment with a non-steroidal aromatase inhibitor	Combination with a steroidal aromatase inhibitor, exemestane	Approval was based on the phase III BOLERO-2 trial (NCT00863655)	[140]
**CDK4/6 inhibitors**						
5	Abemaciclib (2017, 2023)		Refractory HR + /HER2 − metastatic BC	Monotherapy	Approval was based on the phase II MONARCH-1 trial (NCT02747004)	[83–85, 141]
			HR + /HER2 − advanced or metastatic BC	Combination with fulvestrant	Approval was based on the phase III MONARCH-2 trial (NCT02107703)	
			Postmenopausal BC	Combination with a nonsteroidal aromatase inhibitor, letrozole or anastrozole	Approval was based on the phase III MONARCH-3 trial (NCT02246621)	
			HR + /HER2 − , LN + adult patients with early breast cancer at high risk of recurrence	Combination with an adjuvant aromatase inhibitor, tamoxifen	Approval was based on the phase III monarchE trial (NCT03155997)	
6	Palbociclib (2017)		Postmenopausal ER + /HER2 − advanced BC	Combination with letrozole	Approval was based on the phase III PALOMA-2 trial (NCT01740427)	[71, 142]
			HR + /HER2 − metastatic BC after endocrine failure	Combination with fulvestrant	Approval was based on the phase III PALOMA-3 trial (NCT01942135)	
7	Ribociclib (2017)		Postmenopausal advanced BC	Combination with letrozole	Approval was based on the phase III MONALEESA-2 trial (NCT01958021)	[143, 144]
			Postmenopausal advanced BC	Combination with fulvestrant	Approval was based on the phase III MONALEESA-3 trial (NCT02422615)	
			Premenopausal HR + /HER2 − advanced BC	Combination with non-steroidal aromatase inhibitor and goserelin	Approval was based on the phase III MONALEESA-7 trial (NCT02278120)	
**Angiogenesis inhibitors**						
8	Lapatinib (2018)		Postmenopausal HR + , HER2 overexpressing advanced or metastatic BC	Combination with letrozole	Approval was based on the phase III trial (NCT00073528)	[145]
9	Tucatinib (2020)		HER2 + advanced BC	Combination with trastuzumab and capecitabine	Approval was based on the phase III HER2CLIMB trial (NCT02614794)	[146]
10	Neratinib (2020)		HER2 + metastatic BC	Combination with capecitabine	Approval was based on the phase III NALA trial (NCT01808573)	[147]

## Immunotherapy in breast cancer management

The typical management plan for breast cancer involves surgery, radiation, and chemotherapy [29]. However, no optimal chemotherapy has yet been discovered to treat all subtypes of breast cancer. Condensed chemotherapy looks to be more effective than standard therapies; nonetheless, these chemotherapies demand the use of growth factors, which significantly increases the cost of treatment [148, 149]. In addition, the adverse effect of chemotherapeutic drugs is a big challenge in cancer management [150, 151]. Although hormone therapy is a popular non-targeted treatment for breast cancer, it is also associated with serious adverse effects [152]. Targeted therapy, which focuses on specific pathophysiological targets, offers new hope for a more effective and novel therapeutic approach to combat various breast cancer subtypes [152]. However, the efficacy and tolerance of target agents needed to be extensively evaluated in clinical settings. Similarly, immunotherapy has recently been regarded as a potential therapeutic tool to target a particular protein expressed in cancer cells offering enormous hopes for breast cancer treatment.

Despite the fact that breast cancer is not typically a highly immunogenic disease, strategies to modulate the immune system are being tested in clinical settings [153, 154]. Microarray-based investigations of immune-related tumor gene expression showed that the immune signatures influence the clinical outcomes, particularly with HER2 + breast tumors and TNBC [155]. However, immune responses varied significantly according to the subtypes of breast cancer, and it is anticipated that not all breast cancer patients will benefit from the same immunotherapeutic strategy. Thus, beyond the known breast cancer subtypes, it is an important aspect to discover prognostic biomarkers to customize immunotherapies. Programmed death-ligand 1 (PD-L1) is the most recurrently used biomarker even for TNBC. The tumor mutational burden (TMB) is a predictive marker for immunogenicity and foreignness [156]. Tumor-infiltrating lymphocytes (TILs), interferon γ (IFN-γ), programmed cell death ligand-1 (PD-L1), and human leukocyte antigen-I (HLA-I) have also been regarded as predictive markers of immunotherapy.

The presence of negligible effector tumor-infiltrating lymphocytes in the tumor microenvironment forms an obstacle to T-cell-based therapy for certain subtypes of breast cancer. Thus, the development of strategies to improve the number of immune cell infiltration to tumor microenvironment may serve as a potential tool for breast cancer immunotherapy. Breast cancer patients respond well toward immunotherapy in the presence of breast calcifications that are mainly associated with immune dysregulation and Erb-B2 receptor tyrosine kinase 2 (ERBB2) hyperactivation. Induction of hyperthermia in the breast tumor milieu has been regarded as another potential immunotherapeutic approach to directly kill tumor cells [157, 158]. Hyperthermia sensitizes cancer cells toward natural killer (NK) cells and CD8 + cells in a human leukocyte antigen-I (HLA-I) polypeptide-dependent manner. Since estrogen suppresses HLA-I, anti-estrogenic agents could potentiate the action of immunotherapeutic drugs. Thus, HLA-I expression is a key consideration in breast cancer immunotherapy. This section deals with the current developments in immunotherapy for breast cancer, counting immune checkpoint blockades, adoptive T-cell immunotherapy, anti-cancer vaccines, etc.

### Immune checkpoint blockers

**Fig. 5:**
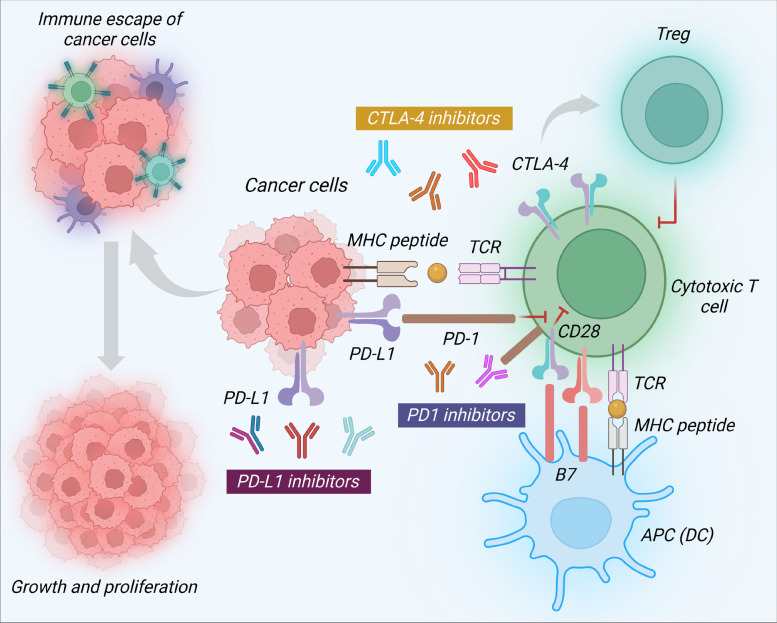
Immune escape mechanism of breast cancer cells and therapeutic role of immune checkpoint blockers in breast cancer treatment. When cytotoxic T-cells in the tumor microenvironment cannot be activated by immunological checkpoints or by the suppressive effect of Tregs, cancer cells are able to withstand the immune assault, survive, and proliferate. CTLA-4 is able to endorse Treg activity leading to an immunosuppressive effect. CTLA-4 binds to B7 (CD80 and CD86) expressed on APCs, such as DCs and inhibits T-cell-mediated immune response. In addition, the binding of CD28 with B7 on APCs suppresses T-cell activity. PD-1/PD-L1 system plays an important role later on and serves to abstract T-cell activity. When PD-1 binds to PD-L1, cytotoxic T-cells become anergic, which further encourages inhibitory signals. APC, Antigen presenting cell; CTLA-4, Cytotoxic T-lymphocytic antigen-4; DC, Dendritic cell; MHC, Major histocompatibility complex; PD-1, Programmed cell death-1; PD-L1. Programmed cell death-1 ligand; TCR: T-cell receptor; Treg, Regulatory T-cell

Immune checkpoints involve both T-cell activation and tolerance. Under normal physiological circumstances, they are essential for preserving immunological homeostasis and self-tolerance. Immune inhibitory signals from tumors may result in an immune escape of tumor antigens. PD-1 (programmed cell death protein-1)/PD-L1 (programmed cell death ligand-1) axis and cytotoxic T-lymphocyte-associated antigen-4 (CTLA-4) are inhibitory signals suppressing the immune response of T-cells [159]. CTLA-4 signaling plays a greater role in preventing the commencement of T-cell response; while PD-1 plays a more significant role later on and serves to abstract T-cell activity in the immunological setting of tumor microenvironment. CTLA-4 binds to CD80 (B7-1) and CD86 (B7-2) expressed on dendritic cells, thus attenuating T-cell-provoked immune reaction. On the other hand, PD-1 activation by PD-L1 persuades inhibition of T-cell immune activity, activation of T-cell death, suppression of pro-inflammatory cytokine production, and induction of antigen tolerance. Inhibiting immune checkpoints by impeding the CTLA-4 or PD-1/PD-L1 axis could therefore reduce the immune escaping of tumor cells and represent a potential immunotherapeutic approach (Fig. 5) [159, 160]. AntiPD-1, anti-PD-L1, or anti-CTLA-4 monoclonal antibodies are among the immune checkpoint blockers being used in clinical practice and some others are still under development. Anti-PD-1 antibodies, such as cemiplimab, pembrolizumab, and nivolumab; anti-PD-L1 antibodies, such as avelumab, atezolizumab, and durvalumab; and anti-CTLA-4, such as tremelimumab and ipilimumab have demonstrated therapeutic potential and were approved for the treatment of various malignancies, including solid tumors [155]. The therapeutic roles of these immune checkpoint blockers based on monoclonal antibodies in breast cancer management have been described in the section below.

#### PD-1/PD-L1 blockers as immunotherapeutic agents

Breast cancer cells especially TNBC cells are abundantly expressing PD-1 and the degree of PD-1 expression is connected to the extent of malignancy [161]. The activation of PD-1 is directly associated with PD-L1. Conventional cancer therapies often endorse PD-L1 activation. A growing body of evidence revealed that blockades of the PD-1/PD-L1 axis stand as a potential therapeutic strategy mainly to treat TNBC. Cemiplimab, pembrolizumab, and nivolumab are some monoclonal antibodies that target PD-1; while avelumab, atezolizumab, and durvalumab target PD-L1.

Cemiplimab is an immunoglobulin G4 (IgG4) monoclonal antibody that targets PD-1 and has been approved by the FDA for the treatment of skin cancer. It has also been found to be efficacious and safe in the clinical setting (NCT03836105) of metastatic cutaneous squamous cell carcinoma [162]. A phase II clinical trial (NCT04243616) is ongoing to check therapeutic responses upon neoadjuvant chemotherapy and cemiplimab treatment in patients with high-risk or progressive HR + and HER2 − breast cancer or TNBC. According to the phase II I-SPY2 trial (NCT01042379), the combination of cemiplimab and a lymphocyte activation gene-3 (LAG-3) inhibitor, fianlimab along with paclitaxel exhibited a significant pathologic complete response than paclitaxel alone in HR + and HER2 − breast cancer or TNBC patients [163].

Pembrolizumab, a humanized monoclonal IgG4 antibody binds to the PD-1 receptor and prevents its communication with PD-L1/2. Pembrolizumab monotherapy (10 mg/kg every two weeks, i.v.) showed a tolerable safety profile in patients with advanced TNBC in the phase Ib KEYNOTE-012 (NCT01848834) study [164]. In the phase II KEYNOTE-086 trial (NCT02447003), pembrolizumab monotherapy also demonstrated potential antitumor efficacy with a manageable safety profile against PD-L1-positive (PD-L1 +) metastatic TNBC [165]. However, the therapeutic response to pembrolizumab is largely dependent on the degree of PD-L1 expression. In the phase III KEYNOTE-119 trial (NCT02555657), even the patients with a PD-L1 combined positive score of ≥ 1 or ≥ 10, pembrolizumab monotherapy for previously treated metastatic TNBC did not increase overall survival significantly in comparison to chemotherapy [166]. Only patients with significantly higher PD-L1 expression responded well to pembrolizumab treatment. Interestingly, some findings proposed that chemotherapeutic drugs can endorse the anti-cancer effects of immune checkpoint blockers through their immunomodulatory responses. In agreement, a phase III KEYNOTE-355 trial (NCT02819518) demonstrated that a combination of pembrolizumab with standard chemotherapy could improve progression-free survival in patients with metastatic TNBC compared to the patients who received only chemotherapy [167]. In the phase Ib/II trial (NCT02513472), combination of eribulin with pembrolizumab was mostly well-tolerated and displayed promising antitumor efficacy in metastatic TNBC patients; however, the effect was found to be largely associated with the presentation of PD-L1 expression [168]. In another phase II study (NCT03222856), the addition of pembrolizumab to eribulin confirmed promising therapeutic efficacy in patients with highly pre-treated, HR + and HER2 − metastatic or locally recurrent breast cancer [169]. In contrast, in an earlier phase II clinical trial (NCT03051659), pembrolizumab plus eribulin did not show any promising clinical outcome in HR + or ERBB2 − metastatic breast cancer patients of either intention-to-treat or PD-L1 + groups compared to only eribulin-treated patients [170]. Furthermore, pembrolizumab plus radiotherapy was found to be safe and efficacious in patients with metastatic TNBC in a phase II clinical setting (NCT02730130) [171]. An ongoing cTRACK-TN trial (NCT03145961) would enlighten the advantages of a watchful increase of pembrolizumab treatment for TNBC patients with measurable circulating tumor DNA.

Nivolumab, a humanized IgG4 monoclonal antibody, is an inhibitor of the PD-1 receptor located at the T-cell surface and prevents its interaction with PD-L1. Nivolumab received the FDA approval for the treatment of melanoma, lung cancer, and renal cell carcinoma [172]. In a phase II TONIC trial (NCT02499367), a significant portion of patients who received the induction treatment with temporary chemotherapy or radiation followed by nivolumab experienced a therapeutic effect that was higher than anticipated in metastatic TNBC patients [173]. In this trial, cisplatin and doxorubicin priming before nivolumab treatment showed a superior therapeutic response, which has been shown to be associated with the immunoregulatory features of these chemotherapeutic drugs to develop favorable tumor microenvironment for PD-1 blockers. Like other immune checkpoint blockers, the therapeutic effects seemed to be linked to PD-1 expression in TNBC despite only a small number of patients representing PD-1 + tumors. However, this finding motivates additional clinical research using nivolumab as a monotherapy or in conjunction with other drugs on breast cancer patients. In contrast, in a phase II study (NCT02834013) comprising 17 patients, a combination of nivolumab with ipilimumab (an anti-CTLA-4 antibody) was found to be associated with extraordinary outcomes in a small subset of patients with chemotherapy-refractory rare metaplastic breast cancer versus no activity [174]. However, in another phase II trial (NCT03316586), cabozantinib plus nivolumab did not demonstrate any promising clinical outcomes in metastatic TNBC patients [175].

Avelumab is a humanized monoclonal IgG1 antibody that targets PD-L1 and prevents PD-1/PD-L1 communication. It endorses NK cell-mediated cytotoxicity and cytokine production to kill tumor cells [176]. In the phase I (NCT01772004) trial, avelumab demonstrated acceptable tolerability and modest therapeutic efficacy in a comprehensively pretreated group of metastatic breast cancer patients [177]. However, a higher possibility of clinical response to avelumab in could be achieved with a higher count of tumor-related immune cells with higher PD-L1 expression specifically in TNBC. In randomized phase III A-BRAVE trial (NCT02926196) is ongoing, which would allow us to interpret the clinical benefits of avelumab among serious TNBC patients. Another phase II trial (NCT03971409) is underway to check the clinical efficacy of avelumab plus liposomal doxorubicin with/without binimetinib or avelumab plus sacituzumab govitecan on patients with stage 4 or inoperable, recurrent TNBC.

Atezolizumab is another ant-PD-L1 monoclonal IgG1 antibody. Atezolizumab monotherapy exhibited acceptable safety with durable therapeutic benefit in metastatic TNBC patients in phase I study (NCT01375842) [178]. Among combination therapy, atezolizumab with nab-paclitaxel exhibited prolongation of recurrence-free survival of metastatic TNBC patients compared to only nab-paclitaxel-treated subgroup in a phase III IMpassion130 clinical setting (NCT02425891) [179]. In the IMpassion130 trial, overall survival for the intention-to-treat population was statistically insignificant, but the PD-L1 + subgroup demonstrated considerable therapeutic effects [180]. Based on IMpassion130 observation, FDA-approved atezolizumab plus paclitaxel therapy for adult patients with locally progressed or metastatic TNBC with tumor-infiltrating immune cells expressing PD-L1. Results from an ongoing trial (IMpassion131, NCT03125902) involving patients with inoperable, previously untreated, locally progressive, or metastatic TNBC may give confirmation of the therapeutic roles of the combination of atezolizumab and paclitaxel. However, the primary observation of the IMpassion131 trial has been disappointing in terms of overall or progression-free survival [181]

Durvalumab, an IgG1 monoclonal antibody, exhibits a strong affinity for PD-L1 binding. In an open-label phase I/II trial (NCT02734004) comprising metastatic breast cancer patients carrying gBRCA mutation, durvalumab (1.5 g every 4 weeks, i.v. infusion) in combination with a PARP inhibitor, olaparib (300 mg, twice daily for 4 weeks) exhibited a favorable antitumor effect with an acceptable safety profile [49]. Durvalumab also exhibited clinical benefits in breast cancer patients when combined with chemotherapeutic drugs. In the phase II GeparNuevo trial (NCT02685059), the inclusion of durvalumab with anthracycline- or taxane-based neoadjuvant chemotherapy to early TNBC patients slightly improved pathological complete response compared to the placebo group; however, the effect was greater for the patients who received durvalumab monotherapy two weeks before the combination therapy [182]. The addition of durvalumab and olaparib to paclitaxel neoadjuvant chemotherapy exhibited better clinical efficacy in a phase II I-SPY2 trial (NCT01042379) over paclitaxel neoadjuvant chemotherapy in HER2 − breast cancer, mainly in serious HR + and HER2 − patients [183]. For patients with stages 1–3 TNBC, durvalumab combined with nab-paclitaxel and dose-dense cyclophosphamide/doxorubicin neoadjuvant chemotherapy led to a 44% pathologic complete response rate in a phase II trial (NCT02489448) [184]. However, the pathologic complete response rate remained higher in PD-L1 + patients. In the SAFIR02-BREAST IMMUNO (NCT02299999) substudy, durvalumab maintenance in metastatic breast cancer patients whose disease remained stable after 6–8 rounds of chemotherapy did not increase progression-free or overall survival in comparison to maintenance chemotherapy. However, maintenance therapy with durvalumab as a single agent improved overall survival in both PD-L1 + and PD-L1 − TNBC patients. These findings support testing durvalumab monotherapy in TNBC patients as maintenance treatment following chemotherapy. The observation anticipated CD274 activation might identify a subset of individuals who would respond well to this anti-PD-L1 antibody; however, further studies are required to reveal the exact reason.

#### CTLA-4 blockers as immunotherapeutic agents

A key suppressive ligand, CTLA-4 is present on effector T-cells [185]. T-cell activation and growth are usually encouraged by CD28 binding, which results in CTLA-4 translocation to and expression on the T-cell surface. Increased surface expression of CTLA-4 following T-cell activation suppresses the effector function of T-cells by inhibiting signaling through the CD28 receptor. The inhibitory signals of CTLA-4 are propagated through the binding of B7-1 (CD80) and B7-2 (CD86) on antigen-presenting cells (APCs). It is worth mentioning that CTLA-4 exhibits higher binding affinity with B7s than CD28. Apart from blocking co-stimulation, CTLA-4 binding blocks IL-2 transcription, which is essential for T-cell growth and NK-based cytotoxic effects. Thus, blocking CTLA-4 by monoclonal antibodies could increase the effector functions of T-cells to kill cancer cells.

Tremelimumab is a CTLA-4-targeted humanized IgG2 monoclonal antibody, which inhibits tumor growth by preventing the interaction between CTLA-4 and B7s and thereby allowing T-cell activation [186]. So far, its clinical efficacy and safety have been assessed in different types of cancer. The FDA approved tremelimumab plus durvalumab combination therapy for inoperable hepatocellular carcinoma. Recently, it received approval from the FDA in combination with durvalumab and platinum-based chemotherapeutic drugs for the treatment of non-small cell lung cancer (metastatic) without EGFR mutation [187]. Although clinical evidence is yet to be available about the effectiveness and safety of pembrolizumab monotherapy in the setting of breast cancer, these issues have been addressed in various combinations comprising pembrolizumab as a component of treatment for breast cancer. In a phase I trial comprising 26 HR + advanced breast cancer patients, pembrolizumab in combination with an aromatase inhibitor, exemestane was well-tolerated with an overall response of ≥ 12 weeks in 42% of patients [188]. Interestingly, CTLA-4 blocking caused immune activation by enhancing inducible co-stimulator activation on CD8 + and CD4 + T-cells. In a pilot study, the combination of tremelimumab with an anti-PD-L1 drug, durvalumab, exhibited poor outcomes in overall metastatic breast cancer patients; however, TNBC patients achieved better therapeutic responses [189]. Surprisingly, TNBC patients who received anti-PD-L1 (durvalumab) monotherapy had a superior response. Pembrolizumab along with radiation therapy showed an acceptable safety profile in a phase I trial even after an extended period [190].

Ipilimumab is a CTLA-4 targeting humanized IgG1 monoclonal antibody, which demonstrates significant anti-tumor activity against a variety of cancers. Preclinical data revealed that ipilimumab promotes the release of IL-2 by TNBC cells to the tumor microenvironment, which endorses the immune response [191]. The FDA approved ipilimumab for the treatment of renal cell cancer, colorectal cancer, malignant pleural mesothelioma, hepatocellular carcinoma, melanoma, and colorectal cancer [172]. In a Phase I trial (NCT01502592), preoperative cryoablation or ipilimumab monotherapy, or their combination in initial stage/operable breast cancer patients exhibited an acceptable safety profile and no delay in surgery was experienced [192]. The outcomes anticipated the possibility of this approach to synergize the antitumor effect through activating anti-tumor immunity. In an open-label and two-stage phase II trial (NCT02834013), nivolumab plus ipilumumab exhibited remarkable responses in 3 out of 17 patients with chemotherapy-nonresponsive, metaplastic breast cancer [174]. This dichotomization with this combination might be associated with specific biomarker/s presented by 18% of patients who received excellent therapeutic benefits and this must be taken into account before executing further trials. Ipilimumab plus nivolumab with neoadjuvant paclitaxel demonstrated prospective overall and pathological complete responses in early-stage TNBC patients in the phase II clinical settings (ACTRN12617000651381) and the observed clinical response was not found to be linked with PD-L1 status [193].

#### Other immune checkpoint inhibitors

Some additional molecules exist that target immunological checkpoints to stop T-cell suppression, such as LAG3, and T-cell immunoglobulin and ITIM domain (TIGIT), or to activate T-cells and boost their cytotoxic function, such as OX-40 (CD134) and 4-1BB [160]. LAG-3 is a novel inhibitory receptor that is exceedingly expressed in regulatory and disabled T-cells. Unlike PD-1/PD-L1 and CTLA-1 blockers, LAG3 blockers can additionally inhibit regulatory T-cells (Tregs) along with endorsing effector T-cell activity. In a phase I trial (NCT00349934), combining the standard chemotherapeutic drug, paclitaxel with the recombinant LAG-3Ig fusion protein, IMP321 showed excellent improvement in objective response rate in patients with metastatic breast cancer in terms of boosting APC, NK cell, and CD8 T-cell counts with excellent safety [194]. A phase IIb AIPAC study (NCT02614833) has been conducted to check the clinical efficacy and safety outcomes of immunotherapy with IMP321 in combination with adjunctive paclitaxel chemotherapy in patients with stage 4 breast adenocarcinoma. The overall outcomes are yet to be published. Several other immune checkpoint targets have been developed, such as B-cell maturation antigen (BCMA), CD19, CD20, CD47, colony-stimulating factor 1 receptor (CSF1R), indoleamine 2,3-dioxygenase (IDO), transmembrane glycoprotein mucin 1 (MUC1), New York esophageal squamous cell carcinoma 1 (NY-ESO1), stimulator of interferon genes (STING), Wilms' tumor gene 1 (WT1), human papillomavirus (HPV), T-cell immunoglobulin domain and mucin domain 3 (TIM3), etc.; however, no specific target is yet to be recognized with promising therapeutic effect in treating breast cancer [195]. Table 2 represents some selected ongoing trials using different immune checkpoint blockers in the context of breast cancer.

**Table 2:** List of some selected ongoing trials with different immune checkpoint blockers in different subtypes of breast cancer

S. no	Phases	Breast cancer subtypes	Immuno-therapeutic agents	Estimated participants	Co-treatment	Recruitment status	Identifiers
**Anti-PD-1/PD-L1 therapy**							
1	I	Advanced TNBC	Pembrolizumab	57	ZEN003694, nab-paclitaxel	Recruiting	NCT05422794
2	I	HLA-A2 + metastatic TNBC	Pembrolizumab	20	PVX-410 vaccine	Active, not recruiting	NCT03362060
3	I	TNBC	Pembrolizumab	Not clearly mentioned	CPI-006	Active, not recruiting	NCT03454451
4	I	Early-Stage TNBC	Pembrolizumab	12	Lenvatinib	Recruiting	NCT04427293
5	I	Metastatic TNBC	Pembrolizumab	11	TTAC-0001	Active, not recruiting	NCT03720431
6	I	TNBC	Pembrolizumab	15	Intraoperative radiation	Recruiting	NCT02977468
7	I (early)	Metastatic TNBC	Pembrolizumab	20	Bortezomib plus cisplatin injections; bortezomib followed by pembro/cis	Recruiting	NCT04265872
8	I	Advanced BC	Pembrolizumab	150	LY3475070	Recruiting	NCT04148937
9	I/II	Locally recurrent or metastatic TNBC	Pembrolizumab	211	Ladiratuzumab vedotin	Recruiting	NCT03310957
10	II	HER-2 − metastatic TNBC	Pembrolizumab	70	Nab-paclitaxel	Active, not recruiting	NCT02752685
11	II	TNBC	Pembrolizumab	26	Paclitaxel, cyclophosphamide, epirubicin	Recruiting	NCT05681728
12	II	TNBC or HR + /HER2 − BC	Pembrolizumab	23	Olaparib	Recruiting	NCT05203445
13	II	HR + localized inflammatory BC	Pembrolizumab	37	Hormonal therapy, radiation	Active, not recruiting	NCT02971748
14	II	HER2 − inflammatory BC	Pembrolizumab	81	EC-paclitaxel	Recruiting	NCT03515798
15	II	HER2 − BC and TNBC	Pembrolizumab	47	Decitabine followed by chemotherapy with cyclophosphamide, paclitaxel, carboplatin	Active, not recruiting	NCT02957968
16	II	TNBC or HR + /HER2 − BC	Pembrolizumab	56	Olaparib, Radiation	Recruiting	NCT04683679
17	II	Anthracycline- refractory TNBC	Pembrolizumab	30	Docetaxel, IL-12 gene therapy	Recruiting	NCT04095689
18	II	Early-stage TNBC	Pembrolizumab	29	AE37 Peptide vaccine	Recruiting	NCT04427293
19	II	TNBC	Pembrolizumab	460	Olaparib, carboplatin, gemcitabine	Active, not recruiting	NCT04191135
20	II	Metastatic TNBC	Pembrolizumab	40	Cyclophosphamide	Active, not recruiting	NCT02768701
21	II	Metastatic TNBC	Pembrolizumab	30	Nac-paclitaxel, carboplatin	Active, not recruiting	NCT03121352
22	II	Inoperable locally recurrent or metastatic TNBC	Pembrolizumab	65	Tavokinogene telseplasmid, immunopulse, nab-paclitaxel	Recruiting	NCT03567720
23	II	TNBC, PD-L1 −	Pembrolizumab	110	Sacituzumab govitecan	Recruiting	NCT04468061
24	II	Metastatic TNBC	Pembrolizumab	87	Carboplatin, Gemcitabine	Recruiting	NCT02755272
25	II	TNBC, BC	Pembrolizumab	12	IRX 2	Active, not recruiting	NCT04373031
26	II	TNBC, ER − , PR − , HER2 − , invasive BC	Pembrolizumab	51	Sacituzumab govitecan	Active, not recruiting	NCT04230109
27	II	TNBC, HR + , HER2 − , LN + BC	Pembrolizumab	120	Radiation Therapy Boost, Paclitaxel, Carboplatin, Cyclophosphamide, Doxorubicin, Capecitabine	Recruiting	NCT04443348
28	III	Inoperable locally recurrent or metastatic TNBC	Pembrolizumab	882	Nab-Paclitaxel, Paclitaxel, Gemcitabine, Carboplatin	Active, not recruiting	NCT02819518
29	I/II	Metastatic TNBC	Nivolumab	51	Romidepsin, cisplatin	Active, not recruiting	NCT02393794
30	I/II	Localized TNBC	Nivolumab	50	Paclitaxel, carboplatin, cabiralizumab	Active, not recruiting	NCT04331067
31	I/II	HER2 +	Nivolumab	390	BDC-1001	Recruiting	NCT04278144
32	II	TNBC	Nivolumab	45	Capecitabine	Active, not recruiting	NCT03487666
33	II	HR − , HER2 − , early stage/resectable BC	Nivolumab	80	Ipilimumab, core biopsy/cryoablation, breast surgery	Recruiting	NCT03546686
34	II	Metastatic TNBC	Nivolumab	52	Cisplatin, doxorubicin (low-dose)	Recruiting	NCT04159818
35	II	TNBC	Nivolumab	84	Radiation, doxorubicin (low-dose), cyclophosphamide, cisplatin	Active, not recruiting	NCT02499367
36	II	Metastatic TNBC	Nivolumab	78	Carboplatin	Active, not recruiting	NCT03414684
37	II	First-line metastatic TNBC	Nivolumab	114	Ipilimumab, capecitabine	Active, not recruiting	NCT03818685
38	II	HR + , HER2 − or TNBC	Cemiplimab	36	Paclitaxel, carboplatin (not mandatory), doxorubicin, cyclophosphamide	Recruiting	NCT04243616
39	II	Different BC subtypes	Cemiplimab	5000	AMG 386 with/without trastuzumab, AMG 479 plus metformin, AMG 386 plus trastuzumab, MK-2206 with/without trastuzumab, T-DM1 and pertuzumab, pertuzumab and trastuzumab, ganetespib ABT-888, neratinib, PLX3397, pembrolizumab (4 cycle), talazoparib plus irinotecan, patritumab and trastuzumab, pembrolizumab (8 cycle), SGN-LIV1A, durvalumab plus olaparib, SD-101 plus Pembrolizumab, tucatinib plus trastuzumab and pertuzumab, cemiplimab, cemiplimab plus REGN3767, trilaciclib with/without trastuzumab plus pertuzumab, SYD985, paclitaxel + dostarlimab encequidar + carboplatin with/ without trastuzumab, paclitaxel + encequidar + dostarlimab with/without trastuzumab, amcenestrant, amcenestrant plus abemaciclib, amcenestrant plus letrozole, ARX788, ARX788 plus cemiplimab, VV1 plus cemiplimab, datopotamab deruxtecan, datopotamab deruxtecan plus durvalumab, zanidatamab, lasofoxifene, Z-endoxifen	Recruiting	NCT01042379
40	I	Different BC subtypes	Avelumab	45	Palbociclib	Recruiting	NCT04360941
41	I/II	TNBC	Avelumab	197	Cyclophosphamide plus JX-594 dose escalation, cyclophosphamide plus JX-594, cyclophosphamide, avelumab plus JX-594 and cyclophosphamide	Recruiting	NCT02630368
42	II	TNBC	Avelumab	150	PF-04518600, utomilumab, binimetinib, sacituzumab govitecan, doxorubicin,	Recruiting	NCT03971409
43	II	BC, metastatic TNBC	Atezolizumab	75	Doxorubicin plus cyclophosphamide, placebo for atezolizumab	Active, not recruiting	NCT03164993
44	II	BC, metastatic TNBC	Atezolizumab	23	Talazoparib, radiation	Active, not recruiting	NCT04690855
45	II	TNBC	Atezolizumab	458	Carboplatin, paclitaxel, epirubicin, cyclophosphamide, surgery	Active, not recruiting	NCT04770272
46	II	TNBC and IV, HER2 − and invasive BC	Atezolizumab	106	Carboplatin	Active, not recruiting	NCT03206203
47	II	TNBC, invasive BC, breast adenocarcinoma	Atezolizumab	37	Nab-Paclitaxel	Active, not recruiting	NCT02530489
48	II	Advanced or metastatic TNBC	Atezolizumab	100	Paclitaxel, bevacizumab		NCT04408118
49	II	TNBC	Atezolizumab	284	Capecitabine or capecitabine (monotherapy)	Recruiting	NCT03756298
50	II	TNBC and Brain Metastasis	Atezolizumab	45	Stereotactic radiosurgery	Active, not recruiting	NCT03483012
51	II	Advanced TNBC	Atezolizumab	52	Stereotactic radiosurgery	Active, not recruiting	NCT03464942
52	III	Inoperable locally advanced or metastatic TNBC	Atezolizumab	180	Nab-paclitaxel	Active, not recruiting	NCT04148911
53	III	TNBC	Atezolizumab	1550	Atezolizumab, paclitaxel (neoadjuvant chemotherapy) carboplatin, doxorubicin, cyclophosphamide, epirubicin, (adjuvant continuation), surgery	Active, not recruiting	NCT03281954
54	III	TNBC	Atezolizumab	2300	Paclitaxel, doxorubicin/epirubicin, cyclophosphamide	Active, not recruiting	NCT03498716
55	III	Recurrent, inoperable locally advanced or metastatic TNBC	Atezolizumab	572	Gemcitabine, capecitabine, carboplatin	Recruiting	NCT03371017
56	III	TNBC	Atezolizumab	278	Carboplatin, nab-paclitaxel, anthracycline chemotherapeutic drug, cyclophosphamide or fluorouracil, epirubicin and cyclophosphamide	Active, not recruiting	NCT02620280
57	I	TNBC	Durvalumab	18	Neoantigen DNA vaccine	Active, not recruiting	NCT03199040
58	I	Stage 2/3 TNBC	Durvalumab	22	PVX-410, Hiltonol	Active, not recruiting	NCT02826434
59	I/II	TNBC	Durvalumab	171	Paclitaxel, carboplatin, oleclumab	Active, not recruiting	NCT02489448
60	I/II	Locally advanced luminal B HER2 −	Durvalumab	57	Paclitaxel, epirubicin, cyclophosphamide	Active, not recruiting	NCT03356860
61	I/II	TNBC	Durvalumab	200	Capivasertib, oleclumab paclitaxel, trastuzumab deruxtecan, datopotamab deruxtecan	Active, not recruiting	NCT03742102
62	II	Advanced and metastatic TNBC	Durvalumab	28	CFI-400945	Active, not recruiting	NCT04176848
63	II	Stage 4 BC AJCC v8, TNBC	Durvalumab	28	Olaparib	Recruiting	NCT03801369
64	II	Stage 4 BC AJCC v8, invasive TNBC	Durvalumab	70	Carboplatin, gemcitabine hydrochloride, nab-paclitaxel, peptide vaccine, poly ICLC, tremelimumab	Recruiting	NCT03606967
65	II	Breast neoplasm	Durvalumab	81	AZD6738, olaparib	Recruiting	NCT03740893
66	II	Different BC subtypes	Durvalumab	5000	Olaparib	Recruiting	NCT01042379
**Anti-CTLA-4 therapy**							
67	II	Stage 4 BC AJCC v8, invasive BC, metastatic TNBC	Tremelimumab	70	Carboplatin, gemcitabine hydrochloride, nab-paclitaxel, peptide vaccine, poly ICLC, durvalumab	Recruiting	NCT03606967
68	I	Different types of cancers including ER − , PR − , HER2 − BC	Ipilimumab	234	XmAb®23,104	Recruiting	NCT03752398
69	I/II	Advanced solid tumors including BC	Ipilimumab	100	Pembrolizumab plus durvalumab	Recruiting	NCT05187338
70	I/II	Advanced solid tumors including metastatic BC	Ipilimumab	914	ONC-392	Recruiting	NCT04140526
71	II	HR − , HER2 − , early stage/resectable BC	Ipilimumab	80	Nivolumab, core biopsy/cryoablation, breast surgery	Recruiting	NCT03546686
72	II	HER2 − metastatic or inoperable BC	Ipilimumab	138	Nivolumab, bicalutamide	Recruiting	NCT03650894
73	I	Advanced solid tumors including TNBC	SI-B003 (a CTLA-4/PD-1 bispecific antibody)	159	-	Recruiting	NCT04606472
74	I/II	Inoperable locally advanced or metastatic cancers including BC	JK08 (CTLA-4 targeting IL-15 antibody fusion protein)	149	-	Recruiting	NCT05620134

*BC* Breast cancer, *CTLA-4* cytotoxic T-lymphocyte-associated antigen-4, *HER2* human epidermal growth factor receptor 2, *HR* Hormone receptor, *PD-1* Programmed death receptor 1, *PD-L1* Programmed death-ligand 1, *PR* progesterone receptor, *TNBC* Triple-negative breast cancer

### Anticancer vaccines

Anticancer vaccines aim at endorsing antigen-specific T-cell-based activation of the immune system to target and eliminate cancer cells [196]. Among different types of vaccines, peptide, protein-based, recombinant DNA, carbohydrate antigen, granulocyte macrophage colony-stimulating factor (GM-CSF)-secreting tumor cell, dendritic cell-based, and dendritic cell-tumor cell fusion vaccines are important in the context of breast cancer [197]. Based on the target, breast cancer vaccines are broadly divided into two categories, such as the vaccines that target HER2 or HER2-associated antigens and the vaccines that target non-HER2-related antigens. The efficacy and safety issues of different HER2 and non-HER2 targeting vaccines in the context of breast cancer under different phases of clinical trials have been discussed in the subsequent section.

#### HER2 targeting breast cancer vaccines

Protein/peptide vaccines that precisely target HER2 are the main focus of developing breast cancer vaccines. E75 (nelipepimut-S, HER2 369–377, or NeuVax), an immunogenic peptide (human leukocyte antigen-A2/A3-constricted) generated from the HER2 protein, can induce a potent anti-HER2 immune response when paired with the immunoadjuvant granulocyte–macrophage colony-stimulating factor [198]. The E75 vaccine has been regarded as a safe and effective immunotherapeutic tool to elicit a peptide-based immune response. In a phase I trial (NCT00841399), it appeared to dramatically lower the risk of cancer recurrence in patients with lymph node-negative (LN–) and HER2 + advanced breast cancer [199]. United States Military Cancer Institute-based trials demonstrated that lymph node-positive (LN +) or LN- breast cancer patients who received an optimal dose of the E75 vaccine experienced similar safety issues to those vaccinated with suboptimal doses; however, exhibited superior HER-2/neu-endorsed immunity to lessen the chance of breast tumor recurrence [200]. However, the study requires a substantially long follow-up to attain conclusive evidence. Final report of phase I/II clinical trials (NCT00841399 and NCT00584789) of the E75 vaccine along with booster immunizations significantly mitigated cancer recurrence in 95.2% of participants with HER2-expressing high-risk breast cancer [201]. Interestingly, the E75 vaccine appeared to be a safe and well-tolerated immunotherapy. Only low-grade local and rare systemic (mild) toxicities were recorded. In a short-term study of a double-blind and randomized phase III trial (NCT01479244) involving patients with LN + and low HER2 + breast tumors, an interim study did not reveal any difference in disease-free survival between E75-vaccinated and placebo-vaccinated patients [202]. Therefore, the trial was suspended at an early phase. A meta-analysis of 24 clinical studies, added to the confusion by revealing that the E75 vaccine significantly reduced disease recurrence but had no meaningful impact on overall survival [203]. Additionally, those who received the E75 vaccine showed moderate variability in their disease-free survival rate.

GP2 (HER2 654–662) is another human leukocyte antigen-A2/3-restricted immunogenic peptide, which is derived from HER2 transmembrane domain. Despite GP2 exhibiting lower affinity toward HLA-A2 compared to E75, however, it could be more immunogenic than E75 [197]. In the United States Military Cancer Institute-based phase I trial, the GP2 vaccine appeared to be well-tolerated and safe, as well as capable of triggering GP2-induced T-cell responses and delayed-type hypersensitivity responses when administered with GM-CSF in the patients with high-risk, LN– breast cancer [204]. This finding encourages additional investigation of the GP2 vaccine to reveal its therapeutic potential to prevent breast cancer recurrence. It has been reported that completion of primary immunization series with GP2 plus GM-CSF entirely stopped cancer recurrence throughout a 5-year follow-up period in HER2/3 + patients, who received trastuzumab treatment following surgery. A phase IIb trial confirmed the outcomes of phase I regarding the safety profile of GP2 plus GM-CSF immunization in women with HER2 + operable breast cancer [205]. This trial demonstrated that the inclusion of GM-CSF did not impart any additional adverse event.

Another peptide vaccine linked to HER2 called AE37 (HER2 776–790) is used as an adjuvant immunotherapy for breast cancer. It is obtained from HER2’s intracellular domain. As a major histocompatibility complex (MHC) class-II epitope, AE37, in contrast to E75 and GP2, primarily endorses CD4 + T cell activation [197]. In the phase I clinical settings, AE37 plus GM-CSF immunotherapy appeared to be well-tolerated and safe, as well as capable of eliciting adjuvant-independent HER-2/neu-mediated immune reaction in disease-free, LN– breast cancer patients [206]. A single-blinded phase II trial (NCT00524277) has been undertaken to compare the efficacy of two HER2 vaccines, viz, GP2 and AE37 in breast cancer patients representing HER2 expression [207]. Both vaccines were found to be safe but effective against only certain subtypes of breast cancer. AE37 vaccination only achieved therapeutic benefit in patients representing low HER2 expressing and TNBC patients; while GP2 vaccination imparted recurrence-free survival only to HER2 overexpressing subgroups. Thus, the extent of HER2 expression is an important marker in selecting peptide-based immunization.

Vaccination with HER2 protein (25, 150, and 900 µg) intracellular domain (amino acids 676–1255) with GM-CSF has been found to elicit a sustained (9–12 months) and dose-dependent HER2-specific T-cell-based and antibody-based immunity in a phase I study involving 29 patients bearing stages 2–4 breast and ovarian cancers with high expression of HER2 [208]. Recombinant-HER2 protein vaccine (20, 100, and 500 µg) in conjugation with an immunostimulant (AS15) also exhibited a satisfactory safety profile in HER2 + breast cancer patients in a phase I trial (NCT00058526). In addition, this vaccine can generate sustained HER2-specific humoral immune responses and prolong overall and disease-free survival [209].

HER2-plasmid DNA vaccination in combination with IL-2, GM-CSF, and trastuzumab treatment exhibited a good safety profile among patients with metastatic HER2 + breast cancer. Although there were no T-cell responses against HER2 immediately following immunization, a significant rise in MHC class-II-mediated T-cell responses against HER2 was found after a specific interval [210]. Vaccination with V930, a DNA vaccine containing plasmid DNA encoding trans-membrane and extracellular domains of human HER2, alone (NCT00250419) or in combination (NCT00647114) with a viral vector vaccine encoding HER2, V932 demonstrated acceptable safety in patients bearing solid tumors; however, cell-based immune response to carcinoembryonic antigen or HER2 was not detected [211]. A couple of trials (NCT00436254 and NCT00393783) with DNA vaccines encoding different forms of HER2-derived proteins are ongoing to measure efficacy and safety issues with them in breast cancer patients.

It has been found that almost one-half of the patients with metastatic TNBC and HER + breast cancers develop brain metastases. Loss of anti-HER2/3 immunity is thought to be associated with this disease progression. A HER2/3 targeting dendritic cell vaccine, alpha-DC1, has been subjected to a phase II trial (NCT04348747) on TNBC and HER2 + breast cancer patients who have brain metastases [212]. After surgery and adjuvant therapy, treatment with autologous dendritic cells pulsed with a HER2 intracellular domain peptide significantly reduced the risk of cancer recurrence in all seven patients in a small clinical setting (NCT00005956) with high HER2 expressing breast cancer (stage II-IV) and six patients showed anti-HER2 antibody [213]. In addition, GM-CSF-secreting HER2 + tumor cell vaccines have been evaluated in clinical trials (NCT00399529 and NCT00093834). These tumor cell vaccines along with chemotherapeutic drugs are safe and capable of imparting anti-HER2 immunity [214, 215].

#### Non-HER2 targeting breast cancer vaccines

Certain subtypes of breast cancer, such as TNBC which represents a lack of ER, PR, and HER2 expressions, provide clinical challenges with HER2-targeting breast cancer vaccines [197]. TNBC expresses a number of non-HER2 tumor-associated antigens, which could be successfully aimed at developing cancer vaccines for clinical applications specifically for TNBC. The cancer-testis antigens (CTAs) may be the most regularly targeted non-HER2 tumor-associated antigens for cancer vaccination [155]. Some notable CTAs, such as New York esophageal squamous cell carcinoma 1 (NY-ESO-1), Wilms' tumor protein (WT1), folate receptor alpha (FRα), melanoma antigen gene protein-12 (MAGE-12), brachyury protein, and p53 could be targeted for developing TNBC vaccines. However, the management of TNBC using the aforementioned CTAs as the targets have not yet shown the anticipated success, and considerable clinical research is required to develop clinically effective CTA-targeting vaccines for TNBC.

Mucin 1 (MUC1) is a protein that is immunologically inaccessible and hyperglycosylated in several epithelial cells; however, an immunologically accessible hypoglycosylated form of this protein is expressed in different malignancies including TNBC. It is thought to be an intriguing tumor-associated antigen for developing TNBC vaccines [216]. Synthetic MUC1 peptide vaccine carrying a toll-like receptor (TLR) 7 agonist has shown preclinical success and raised the prospect for further clinical translation [217]. PANVAC, a recombinant viral vaccine comprises a recombinant fowl pox vector, encoding tumor-associated antigens like carcinoembryonic antigen and MUC1 and another viral vector vaccine containing cancer-associated antigen transgenes as well as different co-stimulatory agent, TRICOM. In a phase II trial (NCT00179309), immunization with PANVAC along with docetaxel treatment to the patients with metastatic breast cancer improved progression-free survival to more than 2 folds over control [218]. Another breast cancer-associated antigen is mammaglobin-A (MAM-A) which is abundantly expressed in 40–80% of breast cancer [197]. In the phase I clinical trial (NCT00807781), the MAM-A DNA vaccine showed satisfactory safety and early signs of improved progression-free survival in metastatic breast cancer patients [219]. Moreover, MAM-A vaccination in breast cancer patients demonstrated activation of inducible co-stimulatory molecules on CD4 + T cells with a substantial reduction of Treg occurrence [220].

Human telomerase reverse transcriptase (hTERT) which plays a key role in oncogenesis, is almost always overexpressed in human malignancies, including breast cancer [221]. Cytotoxic CD8 + T cells can recognize them and endorse the lysis of tumor cells. Thus, hTERT can serve as a cellular immune surveillance target. In a study involving 19 breast cancer patients, the hTERT peptide vaccine was able to elicit CD8 + T cell-based immune response in about 47% of patients [222]. Some phase I trials (NCT01660529, NCT01660529, and NCT02960594) with hTERT are underway.

Tumor-associated carbohydrate antigens are generally regarded as poorly immunogenic, which can be aimed to employ mimicking peptides (mimotopes) to induce anti-human tumor-associated carbohydrate antibodies [223]. Such a mimotope vaccine called P10s-PADRE was subjected to phase II clinical involving metastatic breast cancer patients; however, lack of funding compelled to suspend the patient recruitment. In a phase Ib trial (NCT02229084), 3 weekly immunizations that preceded the first dose of chemotherapy were found to be safe and immunologically promising. However, more trial is required with this combination to address therapeutic benefits [224]. Sialyl-TN (STn) is a carbohydrate epitope, which has been coupled to the keyhole limpet hemocyanin (KLH) carrier protein to develop a vaccine. In a randomized phase II trial, low-dose cyclophosphamide followed for 3 days followed by STn-KLH (100 μg) plus an adjuvant, Enhanzyn™ (DETOX-B) vaccination (on day 0, 2, 5, and 9) to metastatic breast cancer patients exhibited a significantly higher antibody level compared to only vaccine treated group [225]. Even though STn-KLH was well tolerated by patients with metastatic breast cancer, the overall improvement in survival or time to progression has been disappointing in phase III clinical settings. However, in a phase III trial (NCT00003638), STn-KLH in combination with endocrine therapy showed some therapeutic prospects in metastatic breast cancer patients [226]. Additionally, a vaccine that targets the Globo H glycosphingolipid antigen has been clinically studied in patients with different subtypes of breast cancer. In a phase II trial (NCT01516307), treatment with a synthetic Globo H vaccine conjugated with KHL, adagloxad simolenin with adjuvant OBI-821 was well-tolerated and demonstrated anti-Globo H humoral immune responses in patients with Globo H-positive (Globo H +) metastatic overexpressing breast cancer patients. Adagloxad simolenin plus OBI-821 has entered the phase III clinical trial (NCT03562637) to find the efficacy, safety, and tolerability of the vaccine in Globo H + TNBC patients [227].

Even though a number of vaccines have been developed and trialed to improve immunogenicity to different breast cancer subtypes, clinical success has not yet been attained. Thus, substantial research is required to identify the precise target to develop a specific vaccine for the exact subtypes of breast cancer patients.

### Adoptive T-cell therapy

The objective of adoptive T-cell therapy is to endorse cell-based anti-tumor immunity in cancer patients by transferring lymphocytes. Adoptive T-cell therapy includes TIL-based, engineered T-cell receptor (TCR)-based, chimeric antigen receptor (CAR)-based, dendritic cell-based, NK cell-based therapies [228, 229]. It has been regarded as an emerging anticancer immunotherapy achieved by boosting host immunity, particularly in patients representing low immunity. Rosenberg and associates initially introduced the adoptive infusion of autologous TILs as a strategic therapy in 1987. Since then, adoptive TIL therapy has been used in the clinical setting of different types of cancers. However, the therapeutic responses have been heterogeneous based on the cancer types. It has been regarded that recognition of TIL-responsive tumor antigens can improve TIL therapy by endorsing tumor recognition and killing capacity. In the breast cancer setting, it has been revealed that TILs isolated from a specific subtype of tumor can recognize the immunogenic mutations in this tumor milieu and could serve as an immunotherapeutic agent for this specific tumor subtype [230]. In a case report, treatment with TILs reactive against mutant versions of KIAA0368, SLC3A2, CTSB, and CADPS2 proteins in combination with a PD1 blocker and IL-2 demonstrated a durable and complete cancer regression in a patient with chemoresistant, HR + and HER2– metastatic breast cancer [231]. The safety and efficacy of TILs as a monotherapy or in combination with other therapies in the setting of breast cancer may be clarified by the findings of phase I (NCT04111510 and NCT00301730) and phase II (NCT01174121) clinical trials.

CAR T  cell therapy is one of the most recent and effective adoptive T-cell therapy in cancers, which utilizes the specificity of an antibody to target a specific tumor antigen to regulate the cytotoxic capacity of T-cells [229, 232]. Several antigen targets for CAR-T therapy of breast cancer have been discovered through preclinical studies, which include FRα, EGFR, AXL receptor tyrosine kinase (AXL), NKG2D, integrin αvβ3, c-Met, HER2, MUC1, mesothelin, receptor tyrosine kinase-like orphan receptor 1 (ROR1), tumor endothelial marker 8 (TEM8), trophoblast cell-surface antigen 2 (TROP2), etc. [233]. Though TNBC lacks expressions of three major antigens associated with breast cancer, CAR-T therapy still exhibits promising outcomes against the disease. Antigens like MUC-1, NKG2D, AXL, ROR1, c-Met, FRα, mesothelin, etc. have been successfully targeted in various in vitro and in vivo models of TNBC by engineered CAR-T cells [234]. In this context, an encouraging result in the phase I clinical trial (NCT01837602) has already been achieved with c-Met-CAR-T cells. Intratumoral injection of c-Met-CAR-T cells demonstrated an acceptable safety profile in TNBC patients and was found to elicit intratumoral inflammatory response [235]. However, CAR-T cell therapy has also experienced its ups and downs with regard to toxicities and efficacy shortcomings. MUC1-CAR-T cells were mostly studied in early-phase clinical trials (NCT04020575, NCT02587689, and NCT04025216) in TNBC patients. Among them, MUC1-CAR-T cells were mostly studied in early-phase clinical trials (NCT04020575, NCT02587689, and NCT04025216) in TNBC patients. Among them, the NCT04025216 trial has been suspended and the recruitment status of the NCT02587689 trial is unknown. Only the phase I/II trial (NCT04020575) is still recruiting patients to evaluate the safety of MUC1-CAR-T cell therapy. A couple of phase I trials (NCT02580747 and NCT02792114) have been initiated to check the safety of mesothelin-CAR-T cell therapy; however, the results have not been published yet. The phase I trial with NKG2D-CAR-T (NCT04107142) was initiated but the status is unknown, while the phase I trial (NCT02706392) with ROR1-CAR-T (NCT02706392) was terminated. In addition, a few clinical trials had to be abandoned due to unacceptable damage to healthy tissues [236, 237]. In such cases, the target antigen has been observed to be expressed simultaneously by both tumor cells and healthy cells, thus attracting CAR-T cells to both types of tissues consequently resulting in toxicities. A very interesting development to prevent such possibilities has come up in the form of targeting tumor-specific glycoforms of tumor-associated antigens. CAR-T cells have been designed to target aberrantly glycosylated glycoforms of tumor-associated antigens, in addition to the tumor antigens (also expressed by some healthy cells). Certain antigens e.g. Tn, T, and sialyl-Tn are involved in the progression and metastasis of different types of cancers including breast cancer. Underglycosylation of these antigens is a tumor-specific feature owing to the dysregulation of chaperone, Cosmc, and glycosyltransferases [238]. Hence, it is not surprising that targeting these tumor-associated, low-sugar antigens alongside the tumor-specific target antigens by CAR-T cells has expressed futuristic promise to overcome non-selective adverse events. Thus, overcoming the present shortcomings of CAR-T cell therapy might potentially establish it as one of the prime modes of cancer treatment [239].

Additionally, newer prospects for adoptive cell therapy are developing with the discovery of different neoantigens and the application of different immune cells, including NK cells and dendritic cells in cancer management. The clinical results of dendritic cell-based vaccination therapy have been discussed in the earlier section. NK cell-based treatment has not been studied comprehensively in the clinical setting of breast cancer. In only one phase II trial, the adoptive transfer of allogeneic NK cells following lymphodepleting chemotherapy did not demonstrate clinical significance in recurrent breast cancer patients [240]. However, preclinical studies are continuing to develop a successful NK cell-based therapy for breast cancer management. Table 3 represents some selected ongoing trials using adoptive cell therapy in the context of breast cancer.

**Table 3:** List of some selected ongoing trials of adoptive cell therapy in different subtypes of breast cancer

S. no	Phases	Breast cancer subtypes	Precondition	Immuno-therapeutic agents	Estimated participants	Co-adjuvants	Recruitment status	Identifiers
**Tumor-infiltrating lymphocyte (TIL) therapy**								
1	II	Metastatic TNBC	Yes	TIL LN-145	6	-	Active, not recruiting	NCT04111510
2	II	Metastatic cancers including BC (mixed)	Yes	Young TIL	332	Anti-PD1	Recruiting	NCT01174121
**T-cell receptor (TCR) therapy**								
3	II	BC (mixed)	Yes	Sleeping Beauty Transposed PBL		-	Recruiting	NCT04102436
4	II	Metastatic cancers including BC (mixed)	Yes	Autologous TCR-Transduced PBL	270	Anti-PD1	Recruiting	NCT03412877
**CAR-T cell therapy**								
5	I	HER2 + solid tumors including BC	Yes	CCT303-406 CAR-modified autologous T cells (CCT303-406)	15	-	Recruiting	NCT04511871
6	I	Metastatic BC (mixed)	No	huMNC2-CAR44 T cells and huMNC2-CAR44 T cells @ RP2D	69	-	Recruiting	NCT04020575
7	I	Brain or Leptomeningeal metastases from HER2 + cancer including BC	No	HER2-CAR T cells	39	-	Recruiting	NCT03696030
8	I	HER2 + cancer including BC	No	HER2-CAR T cells	45	CAdVEC oncolytic virus	Recruiting	NCT03740256
9	I	Refractory neuroblastoma and other GD2 + cancers including BC	Yes	C7R-GD2.CAR-T cells	94	-	Recruiting	NCT03635632
10	I	Advanced solid tumors including recurrant BC	No	EpCAM CAR-T cells	30	-	Recruiting	NCT02915445
11	I	BC (mixed), metastatic HER2-negative BC	Yes	Mesothelin-targeted T cells	186	-	Active, not recruiting	NCT02792114
12	I/II	BC (mixed)	No	Multi-4SCAR-T cells	100	-	Recruiting	NCT04430595
13	I/II	Relapsed/refractory CEA + cancer including BC	No	CEA CAR-T cells	40	-	Recruiting	NCT04348643
14	I/II	CD44v6 + cancer including BC	No	CD44v6-specific CAR-T cells	100	-	Recruiting	NCT04427449
15	I/II	CD70 expressing cancers including BC	Yes	Anti-hCD70 CAR-transduced PBL	124	-	Recruiting	NCT02830724
16	I/II	Different types of cancer including BC (mixed)	Yes	iCasp9M28z T cell infusions	113	Anti-PD1	Active, not recruiting	NCT02414269
**Dendritic cell (DC) therapy**								
17	I	HER2 + BC	No	HER-2 pulsed DC vaccine	15	-	Active, not recruiting	NCT02063724
18	I	HER2 + BC	No	HER-2 pulsed DC vaccine	7	-	Active, not recruiting	NCT02061423
19	I	HER2 + BC (mixed)	No	DC vaccine (DC1)	31	-	Active, not recruiting	NCT03387553
20	II	HER2 + BC (mixed)	No	DC vaccine (DC1)	119	-	Active, not recruiting	NCT03384914
21	II	BC (mixed)	No	DC-CIK immunotherapy	400	CIK	Active, not recruiting	NCT02491697
22	II	Asymptomatic brain metastasis from TNBC or HER2 + BC	No	Anti-HER2/HER3 DC vaccine	23	Anti-PD1, IFNa2b	Recruiting	NCT04348747
**Natural killer (NK) cell therapy**								
23	I	Advanced or metastatic HER2-expressing solid tumors including BC	Yes	ACE1702	36	-	Recruiting	NCT04319757

*BC* Breast cancer, *CAR-T cell* Chimeric antigen receptor T-cell, *HER2* human epidermal growth factor receptor 2, *PD-1* Programmed death receptor 1, *TNBC* Triple-negative breast cancer

Immune-associated adverse effects with aforementioned immune therapies are mostly unpredictable. Severe endocrine irregularities and albeit rare are the most regular and consistently mentioned adverse reactions with immune checkpoint inhibitors. Despite the toxicities associated with vaccines being acceptable and manageable; they are yet to achieve therapeutic success in breast cancer management. Adoptive cell-based immunotherapy also demonstrated a diverse portrait of adverse events including multiorgan failure, neurotoxicity, cardiac distress, bone marrow challenges and respiratory failure mainly associated with lymphodepletion, immune-mediated side effects including cytokine storm in the clinical setting of breast cancer. Thus, achieving therapeutic success largely depends on careful monitoring pathophysiological status of patients before the execution of immunotherapy or during the course of immune-directed treatment.

## Perspectives

Breast cancer is a heterogeneous pool of diseases that can be divided into various subtypes based on the origin, course, and molecular markers. Protein-gene products that unswervingly influence the biological and clinical traits of cancer cells are prospective targets for the development of novel treatments. Gene signatures have been thought to be the predictors of therapeutic response. Thus, to ascertain appropriate therapy, it is critical to consider the aforementioned factors. In response to chemoresistance and recurrence, scientists have developed targeted drugs for the management of different forms of breast cancer. PARP inhibitors, PI3K/AKT/mTOR inhibitors, CDK4/6 inhibitors and HER2 TKIs emerged as potential targeted therapies of gBRCA-mutated, PIK3CA-mutated, ER + , and HER2 + subtypes of breast cancer, respectively. Some promising clinical outcomes with some of these targeted drugs as monotherapy or in combination with other drugs have been already accredited by regulatory bodies through approval for the treatment of specific subtypes of breast cancer. However, a target-specific therapy could not ensure complete clinical success for a specific subtype of breast cancer. Drug resistance represents a key challenge with current targeted agents. Inclusion of a co-drug that antagonizes drug resistance mechanism and minimizes escape pathway would serve as a potential solution. In this aspect, it is important to ensure that the disease characteristics concerning specific clinical/genetic markers to achieve expected therapeutic success. Thus, it is imperative to ascertain the molecular, genetic, and immune signatures of tumor cells as well as tumor microenvironments before the execution of targeted therapy. Many small molecules have shown promising results in preclinical assays, which may be subjected to further clinical translation. The analyses of cutting-edge approaches, specifically those that prioritize gene/molecular-level analyses of breast cancer subtypes in clinical settings in response to treatment, must be the main focus of upcoming clinical research to reveal a more accurate conclusion. In addition, the adverse reaction to targeted therapeutics must be critically scrutinized, specifically the serious toxicities associated with the treatment require thorough critical interpretation. On the other hand, so far, chemotherapy is the only form of existing treatment for TNBC patients. Extensive clinical development in the field of immunotherapy would expectedly raise new hope in breast cancer management, specifically in TNBC. The introduction of immune-checkpoint blockers and other immunotherapeutic agents to conventional breast cancer therapies enables a higher therapeutic response in terms of progression-free and overall survival including for patients with TNBC. The regulatory body approval of immune-checkpoint blockers in combination with other therapies in TNBC would reveal the prospect of immunotherapy. Moreover, the increasing number of clinical trials demonstrates that the inclusion of immune-checkpoint blockers with other therapeutic drugs would come up as an efficacious therapeutic strategy in breast cancer management especially for TNBC patients. Although the advancement of immune checkpoint blockers is a significant achievement in TNBC treatment, the scope is limited for PD-L1 overexpressing tumors. Thus, more research is required to address this issue. A thorough understanding of tumor subtypes, as well as tumor microenvironments with respect to molecular, genetic, and immune standpoints, would enhance the scope of developing specific immunotherapy directed toward specific breast tumor subtypes to achieve better therapeutic efficacy.

## Conclusion

The recent advancements in targeted therapy have presented a more specific and effective therapeutic option in breast cancer management. Inhibition of specific molecules that promote tumor growth and survival remains the main objective of targeted therapy. Some targeted drugs alone or in combination with other drugs have already got FDA approval for the treatment of different breast cancer subtypes and many of them are under clinical trials. However, drug resistance is a big challenge associated with these drugs. Moreover, the majority of targeted therapy is yet to find clinical success in TNBC patients. On the other hand, immune therapies have emerged as promising targeted therapies specifically for TNBC patients. Some immune-checkpoint blockers in combination with other drugs have already received FDA approval for TNBC treatment. An increasing number of ongoing trials also demonstrates the interest in this field. However, considering the breast cancer heterogeneity, a complete understanding of the molecular, genetic, and immunological landscape of tumor cells as well as tumor microenvironment is the most important aspect to achieving desired therapeutic success with targeted or immune therapy. Thus, it is urgently required to discover/develop specific markers for a clear understanding of breast cancer subtypes to decide on an accurate therapeutic regime.

## Data Availability

Not applicable.

## Abbreviations

AKT: Ak strain transforming
BRCA: Breast cancer gene
CD28: Cluster of differentiation 28
CDK: Cyclin-dependent kinase
CTLA-4: Cytotoxic T-lymphocyte-associated antigen 4
DC: Dendritic cell; EGFR, Epidermal Growth Factor Receptor
ER: Estrogen Receptors
ERK: Extracellular signal-related kinases
FDA: United States-food and drug administration
FGFR: Fibroblast growth factor receptor
HER2: Human epidermal growth factor receptor-2
JAK2: Janus kinase 2
MAPK: Mitogen-activated protein kinase
MEK: Mitogen-activated protein kinase kinase
ERK: Extracellular signal-regulated kinase
mTOR: Mammalian target of rapamycin
NK: Natural killer
PARP: Poly (ADP-ribose) polymerase
PD-1: Programmed death-1
PD-L1: Programmed cell death ligand-1
PI3K: Phosphoinositide 3-kinase
PR: Progesterone receptors
PTEN: Phosphatase and tensin homolog
STAT: Signal transducer and activator of transcription
TILs: Tumor infiltrating lymphocytes
TNBC: Triple-negative breast cancer
